# TFCat: the curated catalog of mouse and human transcription factors

**DOI:** 10.1186/gb-2009-10-3-r29

**Published:** 2009-03-12

**Authors:** Debra L Fulton, Saravanan Sundararajan, Gwenael Badis, Timothy R Hughes, Wyeth W Wasserman, Jared C Roach, Rob Sladek

**Affiliations:** 1 Department of Medical Genetics, Centre for Molecular Medicine and Therapeutics - Child and Family Research Institute, University of British Columbia, West 28th Avenue, Vancouver, V5Z 4H4, Canada; 2 Departments of Medicine and Human Genetics, McGill University and Genome Quebec Innovation Centre, Dr. Penfield Avenue, Montreal, H3A 1A4, Canada; 3 Banting and Best Department of Medical Research, University of Toronto, College Street, Toronto, M5S 3E1, Canada; 4 Center for Developmental Therapeutics, Seattle Children's Research Institute, Olive Way, Seattle, 98101, USA

## Abstract

Unravelling regulatory programs governed by transcription factors (TFs) is fundamental to understanding biological systems. TFCat is a catalog of mouse and human TFs based on a reliable core collection of annotations obtained by expert review of the scientific literature. The collection, including proven and homology-based candidate TFs, is annotated within a function-based taxonomy and DNA-binding proteins are organized within a classification system. All data and user-feedback mechanisms are available at the TFCat portal .

## Abstract 2

TFCat is a catalog of mouse and human transcription factors based on a reliable core collection of annotations obtained by expert review of the scientific literature

## Rationale

The functional properties of cells are determined in large part by the subset of genes that they express in response to physiological, developmental and environmental stimuli. The coordinated regulation of gene transcription, which is critical in maintaining this adaptive capacity of cells, relies on proteins called transcription factors (TFs), which control profiles of gene activity and regulate many different cellular functions by interacting directly with DNA [1,2] and with non-DNA binding accessory proteins [3,4]. While the biochemical properties and regulatory activities of both DNA-binding and accessory TFs have been experimentally characterized and extensively documented (for example, in textbooks devoted to TFs [5,6]), a well-validated and comprehensive catalog of TFs has not been assembled for any mammalian species.

Many gene transcription studies have linked the subset of TFs that bind specific DNA sequences to the activation of individual genes and, more recently, these have been pursued on a genome-wide basis using high-throughput laboratory studies (for example, by performing chromatin-immunoprecipitation) as well as computational analyses (for example, by identifying over-represented DNA motifs within promoters of co-expressed genes). To facilitate such efforts, inventories of TFs have been assembled for *Drosophila *and *Caenorhabditis *species as well as for specific subfamilies of mammalian TFs (Table 1). Since only a limited number of protein structures can mediate high-affinity DNA interactions, collections of TF subfamilies have been constructed using predictive sequence-based models for DNA-binding domains (DBDs) [7-10]. For example, the PFAM Hidden Markov Model (HMM) database [11] and Superfamily HMMs [12] have been applied to sets of peptide sequences to identify nearly 1,900 putative TFs in the human genome [10] and over 750 fly TFs, of which 60% were well-characterized site-specific binding proteins [13]. While these collections have emphasized DNA binding proteins, recent evidence suggests that the contributions of accessory TFs may be equally or more important in establishing the spatio-temporal regulation of gene activity. For example, microarray-based chromatin immunoprecipitation studies have highlighted the key regulatory contributions of histone modifying TFs over the control of gene expression [14]. Therefore, any comprehensive study of TFs must extend beyond a narrow focus of DNA binding proteins to serve as a foundation for regulatory network analyses.

**Table 1:** Transcription factor data resources

Resource	Organism	Reference/URL
Human KZNF Gene Catalog	Human	Huntley *et al*. (2006) [68]/[69]
Database of bZIP Transcription Factors	Human	Ryu *et al*. (2007) [70]/[71]
The *Drosophila *Transcription Factor Database	Fly	Adryan *et al*. (2006) [13]/[72]
wTF2.0: a collection of predicted *C. elegans *transcription factors	Worm	Reece-Hoyes *et al*. (2005) [73]/[74]

The four research laboratories contributing to this report were originally pursuing parallel efforts to compile reference collections of *bona fide *mammalian TFs. In order to maximize the quality and breadth of our gene curation, we combined our efforts to create a single, literature-based catalog of mouse and human TFs (called TFCat). The collection of annotations is based on published experimental evidence. Each TF gene was assigned to a functional category within a hierarchical classification system based on evidence supporting DNA binding and transcriptional activation functions for each protein. DNA-binding proteins were categorized using an established structure-based classification system [15]. A blind, random sample of the functional assessments provided by each expert was used to assess the quality of the gene annotations. The evidence-based subset of TFs was used to computationally predict additional un-annotated genes likely to encode TFs. The resulting collection is available for download from the TFCat portal and is also accessible via a wiki to encourage community input and feedback to facilitate continuous improvement of this resource.

## TF gene candidate selection, the annotation process, and quality assurance

Prior to the initiation of the TFCat collaboration, each of the four participating laboratories constructed mouse TF datasets using manual text-mining and computational-based approaches. As each dataset was created specifically to suit the needs of the research lab that generated it, combinations of overlapping and distinct procedures were applied to collect and filter each dataset (Figure S1 in Additional data file 1). These four, independently established, putative TF datasets laid the foundation for this joint initiative.

To ensure the comprehensiveness and utility of our reference collection, we broadly defined a TF as any protein directly involved in the activation or repression of the initiation of synthesis of RNA from a DNA template. Incorporating this standard, the union of the four sets yielded 3,230 putative mouse TFs (referred to as the UPTF). As complete manual curation of all literature to evaluate TFs is not practical, our curation efforts were prioritized to maximize the number of reviews conducted for UPTFs linked to papers. A manual survey of PubMed abstracts was performed, using available gene symbol identifiers and aliases, to identify genes for which experimental evidence of TF function might exist. Since standardized naming conventions have not been fully applied in the older literature, the associations between abstracts and genes may be incomplete or inaccurate due to the redundant use of the same identifiers for two or more genes. In addition, we did not consider abstracts that made no mention of the gene identifiers of interest or those that, by their description, were unlikely to have conducted transcription regulation-related analyses. From this list of 3,230 putative mouse TFs, coarse precuration identified 1,200 putative TFs with scientific papers describing their biochemical or gene regulatory activities in the PubMed database [16]. The majority of predicted TFs (2,030 of 3,230) had no substantive literature evidence supporting their molecular function. The remaining 1,200 transcription factor candidates (TFCs) were prioritized for expert annotation.

Genes belonging to the TFC set that were associated with two or more papers in PubMed were selected and randomly assigned for evaluation by one or more of 17 participating reviewers. Gene annotations were primarily performed by a single reviewer, with the exception of 20 genes assigned to multiple reviewers for initial training purposes and 50 genes assigned to pairs of reviewers for a quality assurance assessment. In total, 1,058 genes (Table 2) have been reviewed. For each candidate, a TF confidence judgment was assigned (Table 3) based on the literature surveyed. Annotation of each TFC required evidence of transcriptional regulation and/or DNA-binding (for example, a reporter gene assay and/or DNA-binding assay). A text summary of the experimental evidence was extracted and entered by the reviewer, along with the PubMed ID, the species under study, and the reviewer's perception of the strength of the evidence supporting their judgment. Although reviewers were not obligated to continue beyond two types of experimental support, they were encouraged to review multiple papers where feasible. Based on their literature review, annotators were required to classify their determination of each TFC into a positive (TF gene or TF gene candidate), neutral (no data or conflicting data) or negative group (not a TF or likely not). Of the 1,058 TFCs reviewed, 83% were found to have sufficient experimental evidence to be classified either as a TF gene or as a TF gene candidate.

**Table 2:** TFCat catalog statistics

Total number of genes annotated	1,058	100%
Proportion of genes with positive TF judgments	882	83%
Proportion of positive TFs with DNA-binding activity	571	65%
Proportion of DNA-binding TFs that are (double-stranded) sequence-specific	535	94%

**Table 3:** TFCat judgment classifications

Judgment classification	Number of annotations	% of annotations
TF gene	733	61.9
TF gene candidate	256	21.7
Probably not a TF - no evidence that it is a TF	41	3.5
Not a TF - evidence that it is not a TF	30	2.5
Indeterminate - there is no evidence for or against this gene's role as a TF	114	9.6
TF evidence conflict - there is evidence for and against this gene's role as a TF	10	0.8

To simplify data collection and curation, we focused our literature evidence collection and annotation efforts on mouse genes. However, literature pertaining to mouse genes and their human (or other mammalian) orthologs was used interchangeably as evidence for the annotations. Roughly 83% of the annotation literature evidence surveyed was based on a combination of mouse and human data, with roughly equal numbers of papers pertaining to each of these species. Mouse TF genes were associated with their putative human ortholog using the NCBI's HomoloGene resource [16]. With the exception of 40 mouse genes, putative ortholog pairs were matched using defined HomoloGene groups. All but 13 of the remaining 40 were mapped using ortholog relationships in the Mouse Genome Database [17]. Each gene's predicted human ortholog is included in the download data and in the published wiki data.

Depending upon the subset of available papers reviewed for a given TFC, two curators could arrive at different judgments. To ascertain the consistency and quality of our reviewing approach and judgment decisions, we randomly selected 50 genes for re-review and assigned each to a second expert (Tables S1 and S2 in Additional data file 1). Out of the 100 annotations (2 reviews each for 50 genes), 37 paired gene judgments (74 annotations) were concordant and 13 paired gene judgments (26 annotations) were discordant. Examination of the discordant pairs suggested that review of different publications may have produced the disagreement in annotation. To further evaluate this assumption, we extracted a non-quality assurance (non-QA) sample of multiple annotations where different reviewers curated the same genes or gene family members using the same articles (Table S3 in Additional data file 1) and found that these curation judgments were in perfect agreement. Under the assumption that judgment conflicts identified in the QA sample would be resolved in favor of one of the assigned judgment calls, we conclude that 13% of judgments may be altered after additional annotation, suggesting that a system to enable continued review would be beneficial.

Since mouse and human TFs have been evolutionarily conserved among distantly related species [18], we assessed the coverage of our curated TF collection by comparing it with a list of expert annotated fly TFs documented in the FlyTF database [13]. Over half (443 of 753) of the FlyTF genes were found in NCBI HomoloGene groups, producing 184 fly TF-containing clusters that also contained mouse homologs. More than 85% (164 of 184) of these homologous TF genes were in the UPTF set. Inspection of the 20 putative mouse homologs of fly TFs absent from the UPTF set led to the inclusion of 5 genes in both the UPTF and the TFC sets for future curation, while there were no published studies involving the mammalian proteins for the remaining 15 genes. We also assessed TFCat's coverage by comparing it with a classic collection of TFs prepared prior to the completion of the mouse genome [6]. After mapping 506 TFs to Entrez Gene identifiers, we found that 463 were present in the UPTF and 423 were members of the TFC gene list. The remaining 43 genes were added to the UPTF and the TFC list was extended to include 83 additional genes. From these analyses, we conclude that TFCat contains a large majority of known TFs.

## Identification and classification of DNA binding proteins

Genes positively identified as TFs were categorized using a taxonomy to document their functional properties identified in the literature review (Table 4). Notably, 65% (571 of 882) of the genes judged as TFs were reported to act through a DNA binding mechanism and 94% (535 of 571) of these DNA-binding TFs were found to act through sequence-specific interactions mediated by a small number of protein structural domains (Table 5).

**Table 4:** TFCat taxonomy classifications

Taxonomy classification	Number of annotations	% of annotations
Basal transcription factor	39	3.7
DNA-binding: non-sequence-specific	30	2.9
DNA-binding: sequence-specific	591	56.5
DNA-binding: single-stranded RNA/DNA binding	20	1.9
Transcription factor binding: TF co-factor binding	315	30.1
Transcription regulatory activity: heterochromatin interaction/binding	51	4.9

**Table 5:** DNA-binding TF gene classification counts

Protein group	Protein group description	Protein family	Protein family description	Gene count	Predicted occurrences
1.1	Helix-turn-helix group	2	Homeodomain family	131	160
1.1	Helix-turn-helix group	100	Myb domain family	7	16
1.1	Helix-turn-helix group	109	Arid domain family	5	5
1.1	Helix-turn-helix group	999	No family level classification	2	2
1.2	Winged helix-turn-helix	13	Interferon regulatory factor	7	7
1.2	Winged helix-turn-helix	15	Transcription factor family	10	11
1.2	Winged helix-turn-helix	16	Ets domain family	23	23
1.2	Winged helix-turn-helix	101	GTF2I domain family	2	12
1.2	Winged helix-turn-helix	102	Forkhead domain family	26	26
1.2	Winged helix-turn-helix	103	RFX domain family	4	4
1.2	Winged helix-turn-helix	111	Slide domain family	1	1
2.1	Zinc-coordinating group	17	Beta-beta-alpha-zinc finger family	79	450
2.1	Zinc-coordinating group	18	Hormone-nuclear receptor family	43	43
2.1	Zinc-coordinating group	19	Loop-sheet-helix family	1	1
2.1	Zinc-coordinating group	104	GATA domain family	7	12
2.1	Zinc-coordinating group	105	Glial cells missing (GCM) domain family	2	2
2.1	Zinc-coordinating group	106	MH1 domain family	3	3
2.1	Zinc-coordinating group	114	Non methyl-CpG-binding CXXC domain	2	4
2.1	Zinc-coordinating group	999	No family level classification	2	2
3	Zipper-type group	21	Leucine zipper family	41	64
3	Zipper-type group	22	Helix-loop-helix family	71	71
4	Other alpha-helix group	28	High mobility group (Box) family	24	28
4	Other alpha-helix group	29	MADS box family	4	4
4	Other alpha-helix group	107	Sand domain family	3	3
4	Other alpha-helix group	115	NF-Y CCAAT-binding protein family	2	2
5	Beta-sheet group	30	TATA box-binding family	1	2
6	Beta-hairpin-ribbon group	34	Transcription factor T-domain	11	11
6	Beta-hairpin-ribbon group	108	Methyl-CpG-binding domain, MBD family	2	2
7	Other	37	Rel homology region family	10	10
7	Other	38	Stat protein family	6	6
7	Other	110	Runt domain family	3	3
7	Other	112	Beta_Trefoil-like domain family	2	2
7	Other	113	DNA-binding LAG-1-like domain family	2	2
8	Enzyme group	47	DNA polymerase-beta family	1	7
999	Unclassified structure	901	CP2 transcription factor domain family	3	3
999	Unclassified structure	902	AF-4 protein family	1	1
999	Unclassified structure	903	DNA binding homeobox and different transcription factors (DDT) domain family	1	1
999	Unclassified structure	904	AT-hook domain family	3	6
999	Unclassified structure	905	Nuclear factor I - CCAAT-binding transcription factor (NFI-CTF) family	3	3

Members of a DNA-binding TF family share strongly conserved DNA binding domains that, in most cases, have overlapping affinity for DNA-sequences; therefore, a prediction of a TF binding site can suggest a role for the family but does not implicate specific family members. As such, a TF DNA-binding classification system is an essential resource for many promoter sequence analyses in which researchers should prioritize potential trans-acting candidates from a set of equally suitable candidate TFs within a structural class. Capitalizing on large-scale computational efforts for the prediction of protein domains [11,12,19-21], we analyzed each of the TFCat DNA-binding TF protein sequences with the full set of PFAM and Superfamily HMM domain models to predict DBD structures. A total of 20 Superfamily structure types were identified in our set, along with 54 PFAM DBD models (Table S4 in Additional data file 1). Where possible, we linked each double-stranded DNA-binding TF to a family within an established DNA-binding structural classification system [15] that was developed initially to organize the DNA-bound protein crystal structures found in the Protein Data Bank (PDB) [22]. In light of more recent studies, along with a modification of classification requirements (see Materials and methods), an additional set of 16 DBD family classes were added to the system to map domain structures (Table S5 in Additional data file 1).

The DNA binding domain analysis offers some noteworthy observations. The homeodomain-containing genes are prominently represented in our set, comprising 24% (131 of 545) of the classified DBD TFs and 16% of all predicted domain occurrences. The beta-beta-alpha zinc-finger and helix-loop-helix TF families account for 14% (79 of 545) and 13% (71 of 545) of the classified genes, respectively. Given the abundance of zinc-finger proteins in the eukaryotic genomes [23] and recent predictions that this DNA-binding structure makes up a significant portion of all TFs [10], this class may be under-represented. On the other hand, since zinc-finger containing genes are involved in a wide variety of functions, the number of predicted zinc-finger proteins that possess a TF role may be overestimated. In addition, it is likely that certain families of TFs, with central roles in well-studied areas of biology, have been more widely covered in the literature, which may account for the prevalence of literature support for homeodomain TFs.

The majority (392 of 545) of the classified DBD TFs in our list contain a single DNA interaction domain; however, a notable portion (145 of 545) of genes belonging to just a few protein families contain more than one instance of its designated DBD structure. These multiple instances predominantly reside in TFs containing zinc-finger, helix-turn-helix, and leucine zipper domains (Table S6 in Additional data file 1). While most TFs contained single or multiple copies of a single DNA binding motif, our predictions identified eight TFs with two distinct DBDs (Table S7 in Additional data file 1). We removed the second zinc finger-type domain prediction for two of the genes (*Atf2 *and *Atf7*) as this domain is characterized as a transactivation domain in *Atf2 *[24] and may have a similar function in family member *Atf7*. All other predicted gene domains were retained, based on literature that supported their activity or failed to support their removal. Four PFAM DBD models detected in eight proteins are not represented by a solved structure and, therefore, could not be directly appointed in the classification system (see Table 5, Protein group 999). In addition, three nuclear factor I (NFI) proteins were annotated with DNA-binding evidence and predicted to contain a SMAD MH1 DBD. Interestingly, a recent study noted that the DBDs of NFI and SMAD-MH1 share significant sequence similarity [25]. These TFs were also assigned to their own family in the unclassified protein group (Table 5, and Table S5 in Additional data file 1, Protein group 999 and Protein family 905). A group of ten literature-based DNA-binding TFs had no predicted DBDs (Table S8 in Additional data file 1). The absence of detected DBDs may be due, in part, to the limited sensitivity of the models. For example, the *Tcf20 *gene (alias *Spbp*) purportedly contains a novel type of DBD with an AT hook motif [26] that was not predicted by the corresponding AT hook PFAM model. Restricted model representation is also likely the reason for the missing domain predictions of the C4 zinc finger domain in the *Nr0b1 *gene and the basic helix-loop-helix (bHLH) domain in the *Spz1 *gene. Similarly, four DBDs detected with protein group class-level Superfamily models (specifically for zinc coordinating and helix-turn-helix models) could not be further delineated to a protein family level assignment (Table S9 in Additional data file 1), suggesting that their sequences deviate from the family-specific properties represented in PFAM. It is quite possible that there remain to be discovered domains involved in DNA binding by human and mouse TFs.

Most TF DNA-protein interactions occur when the DNA is in a double-stranded state; however, a small number of TF proteins preferentially bind single-stranded DNA [27,28]. We identified in the literature review a set of 16 single-stranded DNA-binding TFs, of which 12 contain HMM-predicted protein domains that are characterized as single-stranded RNA-DNA-binding (Table S10 in Additional data file 1). There may be other DBD TFs in our list that act on both single-stranded DNA and double-stranded DNA but were not classified in the single-stranded DNA DBD taxonomy because this property was not specifically characterized in the literature reviewed. The distinction and overlap between single-stranded DNA and double-stranded DNA binding TFs warrants future attention.

## Generation and assessment of mouse-human TF homology clusters to predict additional putative TFs

Since a transcriptional role can be inferred for closely related TF homologs [7,29-31], researchers interested in the analysis of gene regulatory networks would benefit from access to a broad data collection of both experimentally validated TFs and their homologs. The curated TF gene list was used to identify putative mouse TF homologs in the genome-wide RefSeq collection that have not yet been annotated in our catalog or that were not evaluated because they lack PubMed literature evidence. While sequence homology is often used in preliminary analyses to infer similar protein structure and function, its success may be limited when similar protein structures have low sequence similarity [32] or short homologous protein domains. Based on recent evidence that over 15% of predicted domain families have an average length of 50 amino acids or less [33], we evaluated whether pruning BLAST-derived clusters using a previously published sequence similarity metric [34] could be further improved by explicitly including domain information. Our evaluation of both pruning methods indicated that the inclusion of domain knowledge improved homolog cluster content (Figures S2 and S3 in Additional data file 1). We therefore incorporated both domain structure predictions, using HMMs, and sequence similarity in our homology-based approach to predict additional TF genes.

The homolog prediction and clustering process yielded 227 homolog clusters containing 3,561 genes (3,419 unique genes). The vast majority of the genes (3,284 of 3,561) are associated with only 1 cluster each, although 128 genes were members of 2 clusters and 7 genes were present in 3 clusters. We also identified 72 single gene clusters (singletons), which included 36 TF genes that had only significant BLAST matches to themselves, 12 genes that derived BLAST hits that did not satisfy the homolog candidate cut-offs, 21 genes with cluster members that did not satisfy the pruning criteria, and 3 genes that had no RefSeq model sequence. While our TF-seeded homology inference analysis used cut-offs that likely pruned some false negatives, in an effort to emphasize specificity, it is likely that these singletons represent TFs that share common protein structural features with low sequence similarity.

The curated TF set contains some proteins with properties not commonly associated with TF function. For example, our catalog included the cyclin dependent kinases (*cdk7*, *cdk8*, and *cdk9*), which are reported to directly activate gene transcription (for a review, see [35]). Therefore, the homolog analysis of TFs identified numerous other protein kinases that will likely have no direct involvement in transcription. Similarly, larger clusters seeded by TFs containing other domains not frequently associated with transcription, such as calcium-binding, ankyrin repeats, armadillo repeats, dehydrogenase, and WD40, also attracted false TF predictions.

To assign a quantitative confidence metric for the large clusters of TF predictions, we developed a scoring procedure based on protein domain associations to TF activity annotations from the Gene Ontology (GO) molecular function subtree [36]. The cluster confidence metric was employed using a four-tier ranking system for clusters containing more than ten gene members (42 out of 227 homolog clusters). The majority of these clusters (52% or 22 clusters) received high scores, indicating that they contain a high proportion of TF genes. Given that GO currently annotates only 39% of the TF genes in our catalog in the TF activity node in the molecular function subtree (Table S11 in Additional data file 1), we expect that less frequently occurring protein domains found in small homolog clusters may not yet be represented in GO. Therefore, we did not analyze clusters containing fewer than ten members and we anticipate future refinements in the homolog cluster confidence rankings as TF gene annotation is expanded in GO.

We incorporated our curated set and cluster counts in an analysis to estimate both the total number of TFs and, a smaller subset, the number of double-stranded DNA-binding proteins (see Materials and methods). The cluster counts were adjusted using the observed approximate mean TF (OAMTF) proportions associated with each rank level (Table 6) to account for false positives. From this mouse RefSeq-based analysis, we arrived at an estimate of 2,355 DNA-binding and accessory TFs. Since peptide sequence-dependent analyses can result in both omissions and false predictions of homologous protein structures, readers should regard this figure as a 'best-guess' approximation [32]. A similar analysis conducted over the homolog clusters containing double-stranded DNA-binding TFs resulted in an estimate of 1,510 DNA-interacting TFs. We also performed an extraction of DBD-containing genes from the Ensembl database using the DBDs defined in TFCat. This analysis derived a list of 1,507 putative DNA-binding TFs. These estimates agree well with earlier publications [10,37,38].

**Table 6:** Large cluster ranking criteria

*C*_*n*_	Rank	Implication for unannotated genes in cluster	Fraction of observed approximate mean TFs (OAMTF)
*C*_*n *_≥ 0.20	1	The majority of genes are likely TFs	95%
0.10 ≤ *C*_*n *_< 0.20	2	A higher proportion of genes are likely TFs	75%
0.03 ≤ *C*_*n *_< 0.10	3	A higher proportion of genes are likely not TFs	35%
0.00 ≤ *C*_*n *_< 0.03	4	The majority of genes are likely not TFs	15%

## Maintenance and access of TFCat annotation data

All gene annotations, mouse homolog clusters and human orthologs are published in the TFCatWiki, which is accessible from the TFCat portal. Each wiki article page houses the annotation information for one gene with its content secured against modification. Each gene article page is associated with a discussion page, which is available for comments and feedback by all wiki users. Wiki users can specify that they wish to receive periodic e-mail notification of lists of gene wiki pages and their associated discussion pages that have been updated. Semantic features and functional capabilities are included in the wiki implementation to facilitate easy access to all gene annotation data.

We established a TFCat annotation feedback system workflow process (Figure S4 in Additional data file 1) to encourage continuous improvement of the catalogued gene entries. An issue tracking management system is integrated with the wiki to capture, queue, and track feedback contributions for follow-up by the wiki annotator. Wiki users may view a gene's feedback report summaries and current workflow status through an inquiry made available on each gene's article page. Gene annotation changes, entered through our internally accessible TFCat annotation system, will be flagged and forwarded to the wiki through an automated updating process. Community members who wish to directly contribute to the wiki contents through the backend web application (Figure S5 in Additional data file 1) may contact the authors.

The complete TF catalog resource can be downloaded from our website [39]. The website application enables download of the complete list or a subset of annotated genes by assigned judgment, functional taxonomy, and DNA-binding classification. The data extraction is run real-time against a relational database providing access to the most current TF catalog data.

## Catalog characteristics, comparisons, and utility

The comprehensive catalog of TFs contained in TFCat provides an important resource for investigators studying gene regulation and regulatory networks in mammals. The curation effort assessed the scientific literature for 3,230 putative mouse and human TFs, including detailed evaluation of papers describing the molecular function of 1,058 TFCs, to identify 882 confirmed human and mouse TFs. Each TF was further described within TFCat using a newly developed TF taxonomy. DNA binding proteins, a subset of TFs, were mapped to a structural classification system. As an aide to researchers, an expanded set of putative TFs was generated through a homology-based sequence analysis procedure. Online access to the annotations and homology data are facilitated through a wiki system. An annotation feedback system, linked from the wiki, enables reporting and tracking of community input. An additional website application offers capabilities to extract all or a subset of the catalog data for file download.

For many researchers, the greatest utility of TFCat is the provision of an organized and comprehensive list of DNA binding proteins. The protein-DNA structural classification system used to organize the DBD TFs in the catalog was originally proposed by Harrison [40], further modified by Luisi [41] and extended by Luscombe *et al*. [15]. The DBD analysis and gene/domain counts (Table 5) confirmed that well-known DBD families are represented. The DNA-binding classification system was extended with new family classes to accommodate the majority of predicted DNA-binding structures in our curated TF set (Table 5; Table S5 in Additional data file 1). A new family category was included for unrepresented, double-stranded TF protein-DNA binding mechanisms that were supported by PDB structures or publications. Similar to the analysis and classification performed by Luscombe *et al*. [15], we added structural domain families that were characterized by distinct DNA-binding mechanisms. However, unlike the Luscombe *et al*. approach, we did not consider biological function in our classification decisions. To preserve the properties of the system, the necessary extensions were made within the existing protein groups.

The value in having inventories of TFs has spurred previous efforts to compile collections of DNA-binding proteins. To evaluate the comprehensiveness of our curated collection, we performed a comparison with the gene annotations provided by GO and our DBD classification analysis with domains found in a DBD collection [42]. GO assigns molecular function labels to proteins, including functions falling under the broad category of transcription. The challenge of annotating all genes is daunting and, therefore, it was not a surprise that only 39% (343) of our expert curated collection of TFs has thus far been associated with GO terms linked to transcription (Table S11 in Additional data file 1).

While TFCat is unique in its evidence-based approach to identify mouse and human TFs, there are other compilations of TF binding domain models and predictions of domain-containing proteins. For example, a catalog of sequence-specific DNA-binding TFs (which we will refer to as DBDdb) has been compiled using HMMs to catalog double-stranded and single-stranded sequence-specific DBDs [42]. Comparison of the double-stranded DNA binding subdivision of TFCat with the predictions in DBDdb highlights some key differences between these efforts (Tables S12-S14 in Additional data file 1). For example, the TFCat DNA binding subdivision includes only TFs with published evidence from mammalian studies, whereas the DBDdb collection includes domain predictions based on evidence of sequence-specific DNA binding in any organism. While the two TF resources overlap, they serve complementary purposes. DBDdb is a set of computational predictions generated with protein motif models associated with sequence-specific single or double-stranded binding domains, while TFCat is an expert-curated, highly specific resource that targets the organized identification of all TFs, regardless of DNA binding, in human and mouse. For example, the high mobility group (HMG) domain TFs, which exhibit both specific and non-specific DNA-binding, are excluded from DBDdb but included in TFCat. Moreover, TFCat included only TFs with literature support in mammalian cells, which excludes certain domains included in DBDdb. For example, CG-I has been shown to regulate gene transcription in fly [43] but not in mammals [44].

To complement our large set of curated TF proteins, we conducted a sequence-based homology analysis, propagated from our positively judged TFs, to predict additional TF encoding genes. We applied a confidence ranking metric to predict the number of false positives included in larger homolog clusters (Table 6), which should be considered when extracting un-annotated, predicted TFs. Future adaptations of the TFCat resource could include literature-based judgments of TF homolog predictions. While the homolog clusters as provided are an essential and useful supplement to our evidence-based TF catalog, future predictions may benefit from further structure-based homology research.

Creation of a comprehensive TF catalog provides an important first step in unraveling where, when and how each TF acts. For example, a number of recently published genome-scale studies constructed lists of predicted TFs prior to investigating the spatial and temporal expression characteristics of sets of regulatory proteins [8,9,45,46], in advance of conducting a phylogenetic analysis of genes involved in transcription [47], and as initial input to the analysis of conserved non-coding regions in TF orthologs [48]. The set of literature evidence-supported TFs in TFCat will provide an important foundation for similar future studies.

TF catalogs will become increasingly important and necessary to facilitate the investigation and analysis of TF-directed biological systems. Recent ground-breaking stem cell studies [49,50] have shown the central role of TFs in regulating stem cell pluripotency and differentiation. Understanding the central role of TFs in the control of cellular differentiation has therefore taken on increased importance. Computational predictions in regulatory network analysis of cellular differentiation often highlight a pattern consistent with binding of a structural class of TFs, but fail to delineate which TF class member is acting. TFCat will serve as a reference and organizing framework through which such linkages can progress towards the detailed investigation of candidate TF regulators.

## Materials and methods

### Creation of four independent murine and human TF preliminary candidate data sets

Four TF collections were compiled by four independent approaches. All data sets are available on the TFCat portal.

#### Dataset I

A list of 986 human genes considered 'very likely' plus 913 considered 'possibilities' to code for TFs was manually curated in February 2004 [51] using personal knowledge combined with information in LocusLink (now Entrez Gene), the Online Mendelian Inheritance in Man database (OMIM) [52], and PubMed [16]. Selection was guided by the following definition of a TF: 'a protein that is part of a complex at the time that complex binds to DNA with the effect of modifying transcription'. Inclusion was necessarily subjective for two reasons: the definition of 'transcription factor' is difficult to precisely constrain; and there was not enough information available for many genes to be certain of their function. Genes that primarily mediate DNA repair (for example, *ERCC6*) or chromatin conformation (for example, *CBX1*) were excluded. To be considered, a gene had to have an Entrez Gene entry with a GenBank accession number. Text-based searches for the terms 'transcription factor' or 'homeobox' were used to identify Entrez Gene entries for further analysis. GO node descriptions including the terms 'nucleic acid binding', 'DNA binding', and 'transcription' were used as a supplement to guide gene selection. A total of 998 TFs were present in the set following this initial compilation. After February 2004, periodic additions were made based on new reports in the literature.

#### Dataset II

The objective of this analysis was to identify a comprehensive list of DBDs for TF gene candidate extraction. Firstly, the SwissProt database [53] protein entries (obtained in April 2005) were scanned for descriptors or assigned PFAM [11] and/or Interpro [54] domains (downloaded in April 2005) indicating DNA-binding, DNA-dependent, and transcription. The extracted gene set was then further extended by including SwissProt gene entries that had assignments to the biological process GO node GO:0006355 (regulation of DNA transcription, DNA-dependent) and SwissProt records with text descriptions that included JASPAR database transcription factor binding site class names [55]. A list of unique DBDs was compiled from this extraction. All domains were manually reviewed for evidence strongly suggesting DNA binding and transcription factor activity using both Interpro and PFAM domain descriptions and associated literature references. Domains that did not meet these criteria were pruned from the list. Both known and putative TF genes were extracted from the Ensembl V29 database [56] using the TF DBD PFAM-based list, yielding a set of 1,266 mouse and 1,500 human DNA-binding TF candidates.

#### Dataset III

GO trees were constructed for all mouse and human entries in Entrez Gene by starting with the leaf term from gene2go [36] (downloaded July 19th, 2005) and enumerating all parent terms using file version 200507-termdb.rdf-xml. As we were interested in all genes that could be involved in altering transcription, genes were selected if they had any annotation (including Inferred Electronic Annotations) to GO terms with descriptors 'transcription regulator activity', 'transcription factor activity' and/or 'transcription factor binding' in their tree. We identified 970 mouse genes and 1,203 human genes using this method. As this first extraction did not identify all family members of a putative transcription factor, we performed an additional extraction using the term searches 'DNA binding' and 'transcription factor' against the domain information in the Interpro database [54]. The resulting genes were mapped to Entrez Gene entries using the Affymetrix annotation for the MOE-430 v2 chip. Merging the two lists and removing duplicate entries resulted in 2,131 mouse and 2,900 human candidate genes involved in transcriptional regulation.

#### Dataset IV

We assembled approximately 350,000 isoforms representing approximately 48,000 known and predicted protein-coding mouse genes by mapping seven collections of known and predicted mRNAs to the mouse chromosomes, and clustering them on the basis of overlap (see [57] for source sequences, a representative mRNA from each cluster, and a description of the clustering method). We then assembled 36 known transcription-factor DBDs from PFAM and SMART [58], and screened the approximately 350,000 isoforms using the HMMER software [59] to identify approximately 2,500 known or predicted genes containing at least one of the 36 domains. To map the International Regulome Consortium entries to Entrez Gene, the sequences [60] were compared with RefSeq sequences using BLAST. Only sequences with an expectation value of at most 10^-05 ^were selected and subsequently mapped to Entrez Gene using the Gene2Refseq table.

### Standardizing TF gene candidate annotation

A website annotation tool and MySQL database were developed to standardize and centralize the annotation effort (Figure S5 in Additional data file 1). TF candidate judgments and a high-level taxonomy classification system were established (Tables 3 and 4) for this web-based annotation process. The secure website enables access to only those genes assigned to each annotator. Each gene annotation required input of text summarizing the journal article evidence that, to some degree, supported or refuted the judgment of a gene (or the gene's ortholog in a closely related species) as a TF. One or more PubMed journal articles were summarized in the reviewer comments and a final judgment and general taxonomy classification were assigned.

Ten trial genes, randomly selected from the list of TFCs, were assessed by four reviewers. The set of annotations for each trial gene was evaluated for literature evidence selected and annotation content and formatting. This evaluation was used to develop annotation evidence guidelines and a suggested general documentation format for the annotation process, which was included in the annotator help guidelines.

### Selection and annotation of a subset of TF candidates

The mouse TF candidate datasets were merged, using mapped NCBI Entrez Gene identifiers, into a single non-redundant dataset. Gene2PubMed file counts were extracted and merged by Entrez Gene ID. Genes were manually pre-curated for evidence supporting TF activity by scanning NCBI PubMed abstracts (where available) using both standard gene symbols and aliases and examining GeneRIF entries for each gene in the dataset. Genes with literature evidence suggesting TF function were included in the list of TFCs to be annotated. A set of TFCs associated with two or more PubMed abstracts (based on Gene2Pubmed data and excluding the large annotation project articles) were extracted from the TFC list and randomly assigned to each of 17 reviewers based on pre-determined reviewer allocation counts. Each TFC was reviewed and judged by the assigned reviewer for TF evidence in the literature as described above. We also extracted and entered the PubMed information accompanying 22 TF DNA-binding profiles from the JASPAR database [55].

During this research project, the Entrez Gene numbers were maintained using the NCBI Gene History file. TFCat gene identifiers were maintained (changed or merged or deleted) if a corresponding change was recorded in this file.

### Randomly sampled quality assessment and auditing of TF annotations

TF gene candidates were randomly selected from each reviewer-assigned gene set based on the assigned proportions across all reviewers to form a list of 50 genes for annotation QA testing. Each gene was allocated to two reviewers for annotation in a blind QA test. The QA gene annotations were extracted and reviewed for TF judgment and taxonomy classification consistency. A second round of annotation auditing was performed to ensure consistency in the recorded annotation data. All annotations were examined for alignment of PubMed evidence reviewed and assigned judgment and functional taxa. Misaligned annotations were forwarded to the annotator for review and revision.

### TFC quality assurance comparisons

To assess sensitivity (coverage) in our initial curated TF list, we compared our gene set with TF genes identified in two TF collections. Approximately 800 gene symbols listed in a TF textbook index, authored by Joseph Locker [6], were manually reviewed and mapped, where possible, to 506 mouse Entrez Gene identifiers using gene descriptions and citations provided in the text. A TF comparison was also performed against the list of annotated fly TFs found in the FlyTF database [13] by mapping, where possible, FlyBase identifiers to NCBI gene identifiers to locate their corresponding mouse homolog in a HomoloGene group [16].

Upon completion of the TFCat curation phase, we performed comparisons with GO [36] and the DBD Transcription Factor Prediction Database resource [42]. To compare our curated set with GO, we developed software to enumerate the number of our TF genes in the GO molecular function subtree under the 'transcription regulator activity' node. We used the Mouse Xref file found in the GO Annotation Database [61] to map the TF Entrez gene numbers to the gene identifiers available in the GO database. The DBD resource comparison involved downloading the mouse (*Mus musculus *49_37 b) and human (*Homo sapiens *49_36 k) predicted TF sets and development of software to extract all DBD models identified in those records. We then compared the domains found in the DBD mouse/human set with those domain models annotated as DNA-binding in our curated TF set.

### Human-mouse ortholog assignment

Human-mouse predicted orthologs were assigned using NCBI HomoloGene groups [16] with one-to-one relationships between the mouse and human genes. Those few genes that did not have a one-to-one relationship were manually inspected and, when available, a preference was given to the human non-predicted RefSeq gene model or an assignment was made using the closest Blast alignment scores between a mouse and human gene pair. Where HomoloGene entries were not available for both human and mouse, ortholog assignments identified in the Mouse Genome Database were used.

### TF DNA-binding structure analysis and classification

A DNA-binding protein classification system, an extension of the work from Luscombe *et al*. [15], was utilized to classify all genes judged as TFs with DNA-binding activity. Structural assignments were made utilizing the HMMER software to enumerate a full set of Superfamily (SCOP-based) HMMs [12] with a threshold of 0.02 and PFAM HMMs [11] for each gene using gathering threshold cut-offs and a calculated model significance value ≤ 10^-2^. The Superfamily domain sequences predicted in the TF gene set were subjected to a PFAM HMM analysis to identify PFAM domain models that are satisfied by the same sequences (Table S4 in Additional data file 1). Both redundant and non-redundant models were then mapped to the DNA-binding structure classification using model structural descriptions and based on review of related literature for PDB entries that contain these domains.

The DNA-binding classification was extended with additional family classes to accommodate the predicted DNA-binding structures encountered in the curated set of DBD TFs (Table 5; Table S5 in Additional data file 1). To evaluate the structural similarity of DBDs, we performed alignments using the protein structure comparison web tool Secondary Structure Matching (SSM) [62]. We identified PDB entries for each of the new DBD families, with a preference for DNA-bound structures. The DBD chains of each PDB entry were aligned with the entire PDB archive (incorporating lowest acceptable matches of 40% and defaulting the remaining parameters) to identify similar DBD structures based on Q-score metric clustering results. A new protein family classification was established if the structure aligned only to itself or was clustered (by Q-value) within its own set of family class structures. In a few cases, where a structure aligned reasonably well with another family in the classification system, PubMed articles were consulted to derive a final decision and any borderline cases were noted and described in the family class description text (Table S5 in Additional data file 1). Each DNA-binding TF was then assigned to one or more DNA-binding families in the classification system if it was predicted to contain the related DBD structure.

### Identification of homolog sets for mouse TF genes

A homolog analysis process was implemented that considers both sequence similarity and predicted protein domain commonality, and uses a computationally simplified clustering approximation, loosely motivated by proportional linkage clustering [63]. We initially identified sequence similarity using BLASTALL [64] analysis over a full mouse protein RefSeq [65] dataset with an expect value cut-off of 10^-3 ^and enumerated all HMM PFAM domains over an extracted full representation of the mouse genome using NCBI RefSeq sequences. To extract putative homolog candidates for each TF gene, we incorporated a metric, originally proposed by Li *et al*. [34], which considers the ratio of aligned sequence length to the entire length of each sequence. Given the focus on mouse genes, the formula for this metric, which we will refer to as metric *I'_s_* , was revised to utilize sequence similarity rather than identity. Our metric is computed as:

$${I^{\prime }}_{s}=S\times Min(n_{1}/L_{1},n_{2}/L_{2})$$

- where S is the proportion of similar amino acids (as defined by the Blosom62 matrix) across the hit, *L*_*i *_is the length of sequence *i *(*i *is the query or hit sequence), and *n*_*i *_is the number of amino acids in the aligned region of sequence *i*. We considered only homolog candidates that had a maximum hit significance of 10^-4 ^and allowed for a high level of sensitivity by requiring that the computed *I'_s_* values were at least 0.06. We did not include any genes that had been reviewed and deemed not TFs.

Our survey of a set of TF gene family sequence characteristics suggested that some known DBDs were contained in a small fraction of the total TF protein sequence. However, similarly short alignments between a TF gene and other hit sequences (low *I'_s_* values) can yield a significant amount of false positives. We used well-documented SRY-related HMG-box transcription factor (Sox) and Forkhead transcription factor (Fox) TF families (Tables S15 and S16 in Additional data file 1) to evaluate two cluster pruning strategies and selected an approach that increased cluster specificity (proportion of members of a test set in a cluster) without decreasing cluster sensitivity (number of cluster members that are members of a test set). To evaluate cluster pruning of the Blast-based clusters using strictly an *I'_s_* threshold method, we computed cluster sensitivity and cluster specificity over an increasing range of *I'_s_* values, using the Sox and Fox validation sets (Figures S2 and S3 in Additional data file 1). An *I'_s_* value was computed between the query sequence and every member in the cluster and a member (gene) was pruned if the *I'_s_* did not satisfy a cut-off threshold. Cluster sensitivity and cluster specificity were computed for the range of *I'_s_* values and compared. We then assessed a second cluster pruning approach over a successive range of *I'_s_* values requiring that all predicted domains in a cluster member (gene) match the query gene or, when this criteria could not be met, a particular *I'_s_* value threshold be satisfied (Figures S2 and S3 in Additional data file 1). Inclusion of a domain-based method as a primary criteria for pruning with the incorporation of a stricter *I'_s_* value criteria when the domains did not match, in most cases, maintained cluster sensitivity while preserving or improving cluster specificity. Importantly, higher cluster sensitivity and cluster specificity levels enabled comprehensive Sox HMG and Fox Forkhead families to emerge when we applied a proportional linkage clustering approximation approach to merge the overlapping clusters (Figures S6 and S7 in Additional data file 1). While the sole application of an *I'_s_* value as a pruning criteria may not generate comprehensive TF family clusters (compare panel B in Figures S6 and S7 in Additional data file 1), our analyses suggested that this metric on its own, implemented with higher parameter values, is useful for identifying closely related subfamily members (Figure S8 in Additional data file 1). Motivated by these assessment results, we implemented a cluster pruning step that required that either all predicted PFAM enumerated domains in the TF gene be matched in a homolog candidate or that the *I'_s_* value between the query TF gene and its homolog hit be no smaller than 0.21 with a sequence similarity no less than 30%. This resulted in 830 overlapping sets consisting of 48,555 members in total.

To cluster and merge the sets, we implemented a method that considers a proportional linkage median-based relationship between sets. The algorithm performed iterations of set merges, combining two sets S and T if at least half of the genes in the smaller set matched genes in the larger set, that is, if there were |(min(|*S*|,|*T*|))/2| matching genes. To mitigate the cluster attraction strength properties of initially larger and possibly noisier clusters, the merge process iteratively considered and executed merging over smaller to progressively larger cluster cardinalities using increments of 10. Cluster membership attained a steady-state convergence within 700 iterations.

A cluster confidence metric was developed to measure the number of potential false positives in a large (cardinality > 10) homolog cluster using predicted domain content. We mapped the mouse genes with the enumerated PFAM domains to terms in the GO molecular function subtree. We tallied the number of times a specific domain is contained within a gene annotated to the transcription regulator activity node and its child nodes versus the number of times the domain is found in a gene annotated to some other activity node to compute a probability of a particular domain *P*_*d *_being associated with TF function. The majority of GO annotation evidence codes were included, with the following exceptions: IEA (Inferred from Electronic Annotation), ISS (Inferred from Sequence or Structural Similarity), and RCA (Inferred from Reviewed Computational Analysis). To evaluate cluster confidence *C*_*n*_, we first enumerated the number of genes that contain a specific domain within a cluster *C*_*d *_and the number of genes in each cluster *C*_*g *_to weight a domain's association to TF activity:

$$N_{d}=\frac{C_{d}}{C_{g}}P_{d}$$

- and, secondly, included those cluster domains that satisfy *D *= {*C*_*d *_≥ ⌈*C*_*g*_/4⌉} to compute *C*_*n*_, using the following equation:

$$C_{n}=\frac{\sum_{i\in D}N_{d_{i}}}{\left|D\right|}$$

All cluster confidence values and cluster membership were reviewed and qualitatively assessed based on the proportion of verified TFs and binned into four partitions with associated confidence rankings (Table 6).

To derive an estimate for the total number of TFs in the human and mouse species, we computed the number of known and predicted TF homologs and adjusted this amount by the cluster rank OAMTF (Table 6) to obtain a prediction of 2,355 DNA-binding and accessory TFs. To obtain a ballpark figure for a total number of DBD TFs, we performed a separate homolog clustering analysis seeded by genes curated with double-stranded DNA binding activity and reduced the counts using the OAMTF proportions by cluster rank, where applicable. The homolog-based analysis generated an estimate of 1,510 DBD TFs. To support our DBD homology-based count analysis, we developed PERL scripts to query the mouse Ensembl mus_musculus_core_47_37 and ensembl_mart_47 databases for extraction of predicted DNA-binding TFs using the identified PFAM DBDs in TFCat. This extraction produced a total of 1,507 Ensembl mouse genes (1,416 records supported by Mouse Genome Informatics (MGI); 23 RefSeq and Entrez Gene sourced records; 29 Uniprot/SPTREML predicted genes; and 39 Ensembl predicted gene models).

### Website download access, wiki publication and annotation feedback

The MediaWiki software was used to implement the TFCatWiki, with some modifications and additions made to the base software code and configuration files. We included the Semantic MediaWiki [66] extension to facilitate access and searching. Each article page contains the annotation information for one gene and has been configured to disallow edits, although enabling all associated discussion pages for contribution. Software was developed to extract data from the TFCat wiki database to create the wiki pages.

We implemented a feedback tracking function using the MantisBT software system [67], a well-established, open-source, issue monitoring system, to accommodate tracking and follow-up management of TFCat feedback contributions. PHP interfaces and software were developed to populate MediaWiki user information to the feedback system and provide direct query access to feedback records by gene. We also integrated new data update flagging mechanisms into our internally available TFCat annotation software tool to identify new or modified gene annotation information that requires re-population to the gene wiki page.

The MediaWiki software includes a Watch function, which issues individual e-mails when information is changed on a wiki page by a wiki user. We developed an e-mail feature that optionally provides lists of wiki pages that have been changed via the backend auto-update process. To enable this feature, we developed an external PHP program (MediaWiki) hook and an associated MySQL database table to solicit user entry and capture of desired e-mail parameter options and notification frequency. An e-mail notification process was developed that issues e-mails for wiki content updates based on user-selected parameters.

## Abbreviations

DBD: DNA-binding domain; DBDdb: DBD Transcription Factor Database; Fox: Forkhead transcription factor family; GO: Gene Ontology; HMG: high mobility group; HMM: hidden Markov model; NFI: nuclear factor I; OAMTF: observed approximate mean TF; PDB: Protein Data Bank; QA: quality assurance; Sox: SRY-related HMG-box transcription factor family; TF: transcription factor; TFC: transcription factor candidate; UPTF: union of putative TFs.

## Authors' contributions

Initial putative TF datasets were created by JR (dataset I), DLF (dataset II), SS (dataset III), and GB (dataset IV). SS created the merged dataset and performed an NCBI mapping for dataset IV. DLF designed, implemented, and populated the centralized TFCat database and annotation website tool. SS provided some text data extractions for the TFCat database. RS and DLF precurated the unified dataset. JR, RS, DLF, SS, GB, TH, and WWW acted as the core group of gene annotators. DLF performed the TF reference collection comparisons. Annotation audits were performed by DLF, WWW, RS, and SS. DLF established and implemented the structural classification mapping methodology and performed the analysis of DNA-binding structures to extend the DNA-binding structural classification. DLF devised and implemented the homolog analysis and gene clustering process. DLF, SS, and RS worked on the wiki gene page format. DLF designed, developed and implemented the wiki. DLF developed and implemented the website TFCat data download portal. WWW, JR, and RS provided co-supervision for this project, with the implementation led by DLF. DLF wrote the draft of the manuscript, with further modifications and edits contributed by WWW, RS, JR and SS. All authors read and approved the final manuscript.

## Additional data files

The following additional data is available with the online version of this paper: a PDF that includes Tables S1-S16 and Figures S1-S8 (Additional data file 1).

## Supplementary Material

Additional data file 1 Table S1: gene annotation judgment summary counts from the quality assurance assessment process. Table S2: quality assurance gene pair judgment annotations. Table S3: independent annotations of TFs when the same PubMed evidence was used. Table S4: PFAM and Superfamily group model DBD predictions for the annotated TF genes. Table S5: DNA-binding classification extensions added to the Luscombe *et al*. [15] classification system. Table S6: protein class counts of genes predicted to contain multiple instances of the same DBD. Table S7: DNA-binding TFs predicted to contain two different DNA-binding classes. Table S8: DNA-binding TFs that do not contain a detected DBD. Table S9: DNA-binding TFs with no detected protein family-level domain. Table S10: annotated single-stranded DNA-binding TFs. Table S11: summary of the counts enumerated in the TFCat comparison with GO. Table S12: summary of the counts enumerated in the comparison of TFCat classified HMM DBDs with the DBD database (DBDdb). Table S13: Superfamily DBD comparisons with DBDdb. Table S14: PFAM DBD comparisons with DBDdb. Table S15: Fox family test set genes. Table S16: Sox family test set genes. Figure S1: Venn diagram of the overlap of the four initial TF datasets. Figure S2: plots for the analysis of cluster pruning methods using the Fox test set. Figure S3: plots for the analysis of cluster pruning methods using the Sox test set. Figure S4: TFCat annotation workflow implementation. Figure S5: screen shots of the backend web-based TFCat annotation tool. Figure S6: Sox-containing cluster membership for the evaluated cluster pruning methods. Figure S7: Fox-containing cluster membership for the evaluated cluster pruning methods. Figure S8: an example of pruned Fox-containing clusters generated using the *I'*_*s *_only method using a threshold of 0.21. Click here for file
