# Supplementary material for: TFCat: the curated catalog of mouse and human transcription factors
Source: Genome Biol. 2009 Mar 12;10(3):R29. doi: 10.1186/gb-2009-10-3-r29 (PMC2691000; doi:10.1186/gb-2009-10-3-r29)

Additional Data File 1: Tables

Table S1. Quality assurance gene annotation judgment summary

	**Gene Annotation Pairs**
Concordant Judgments	37
Discordant Judgments	13
# QA Gene Annotations	50

Table S2. Quality assurance gene annotation pair judgments

Gene ID	Description	Reviewer	Judgment
12022	BarH-like homeobox 1	Jared Roach	TF Gene Candidate
12022	BarH-like homeobox 1	Elodie Portales-Casamar	TF Gene Candidate
12455	cyclin T1	Jared Roach	Not a TF
12455	cyclin T1	Debra Fulton	TF Gene Candidate
12590	caudal type homeo box 1	Sarav Sundararajan	TF Gene
12590	caudal type homeo box 1	Rob Sladek	TF Gene
14247	Friend leukemia integration 1	Gwenael Breard	TF Gene
14247	Friend leukemia integration 1	Elodie Portales-Casamar	TF Gene
15375	forkhead box A1	Gwenael Breard	TF Gene
15375	forkhead box A1	Rob Sladek	TF Gene
15384	heterogeneous nuclear ribonucleoprotein A/B	Sarav Sundararajan	TF Gene
15384	heterogeneous nuclear ribonucleoprotein A/B	Rob Sladek	TF Gene
16917	LIM homeobox transcription factor 1 beta	Jared Roach	TF Gene
16917	LIM homeobox transcription factor 1 beta	Wyeth Wasserman	TF Gene Candidate
17300	forkhead box C1	Jared Roach	TF Gene
17300	forkhead box C1	Elodie Portales-Casamar	TF Gene
17702	homeo box, msh-like 2	Wyeth Wasserman	TF Gene
17702	homeo box, msh-like 2	Shannan HoSui	TF Gene
18504	paired box gene 2	Jared Roach	TF Gene
18504	paired box gene 2	Shannan HoSui	TF Gene
18508	paired box gene 6	Jared Roach	TF Gene
18508	paired box gene 6	Tim Hughes	TF Gene
18854	promyelocytic leukemia	Jared Roach	TF Gene Candidate
18854	promyelocytic leukemia	Elodie Portales-Casamar	Not a TF
19013	peroxisome proliferator activated receptor alpha	Gwenael Breard	TF Gene
19013	peroxisome proliferator activated receptor alpha	Sarav Sundararajan	TF Gene
19698	avian reticuloendotheliosis viral (v-rel) oncogene related B	Jared Roach	TF Gene
19698	avian reticuloendotheliosis viral (v-rel) oncogene related B	Wyeth Wasserman	TF Gene
19708	D4, zinc and double PHD fingers family 2	Rob Sladek	Indeterminate
19708	D4, zinc and double PHD fingers family 2	Wyeth Wasserman	TF Gene Candidate

Table S2. Quality assurance gene annotation pair judgments (continued)

Gene ID	Description	Reviewer	Judgment
20182	retinoid X receptor beta	Andrew Kwon	TF Gene
20182	retinoid X receptor beta	David Martin	TF Gene Candidate
20466	transcriptional regulator, SIN3A (yeast)	Gwenael Breard	TF Gene Candidate
20466	transcriptional regulator, SIN3A (yeast)	Debra Fulton	TF Gene
20588	SWI/SNF related, matrix associated, actin dependent regulator of chromatin, subfamily c, member 1	David Martin	TF Gene Candidate
20588	SWI/SNF related, matrix associated, actin dependent regulator of chromatin, subfamily c, member 1	Shannan HoSui	TF Gene
20613	snail homolog 1 (Drosophila)	Rob Sladek	TF Gene
20613	snail homolog 1 (Drosophila)	Wyeth Wasserman	TF Gene Candidate
20630	U1 small nuclear ribonucleoprotein 1C	Jared Roach	Not a TF
20630	U1 small nuclear ribonucleoprotein 1C	Shannan HoSui	TF Evidence Conflict
20669	SRY-box containing gene 14	Wyeth Wasserman	TF Gene Candidate
20669	SRY-box containing gene 14	Mark Minie	TF Gene Candidate
20671	SRY-box containing gene 17	Wyeth Wasserman	TF Gene Candidate
20671	SRY-box containing gene 17	Elodie Portales-Casamar	TF Gene
21339	TATA box binding protein (Tbp)-associated factor, RNA polymerase I, A	Sarav Sundararajan	Not a TF
21339	TATA box binding protein (Tbp)-associated factor, RNA polymerase I, A	Debra Fulton	TF Gene
21387	T-box 4	Sarav Sundararajan	TF Gene Candidate
21387	T-box 4	Rob Sladek	Indeterminate
21416	transcription factor 7-like 2, T-cell specific, HMG-box	David Martin	TF Gene
21416	transcription factor 7-like 2, T-cell specific, HMG-box	Andrew Kwon	TF Gene
21685	thyrotroph embryonic factor	Gwenael Breard	TF Gene
21685	thyrotroph embryonic factor	Debra Fulton	TF Gene
21833	thyroid hormone receptor alpha	Jared Roach	TF Gene
21833	thyroid hormone receptor alpha	Debra Fulton	TF Gene
22032	Tnf receptor associated factor 4	Debra Fulton	TF Gene
22032	Tnf receptor associated factor 4	David Martin	Indeterminate
22608	Y box protein 1	Wyeth Wasserman	TF Gene
22608	Y box protein 1	David Martin	TF Gene

Table S2. Quality assurance gene annotation pair judgments (continued)

Gene ID	Description	Reviewer	Judgment
22634	pleiomorphic adenoma gene-like 1	Jared Roach	TF Gene
22634	pleiomorphic adenoma gene-like 1	Debra Fulton	TF Gene
22666	zinc finger protein 161	Sarav Sundararajan	TF Gene
22666	zinc finger protein 161	Wyeth Wasserman	TF Gene
22718	zinc finger protein 60	Rob Sladek	TF Gene Candidate
22718	zinc finger protein 60	Tim Hughes	TF Gene Candidate
22722	zinc finger protein 64	Wyeth Wasserman	TF Gene Candidate
22722	zinc finger protein 64	Elodie Portales-Casamar	Indeterminate
56233	histone deacetylase 7A	Wyeth Wasserman	TF Gene Candidate
56233	histone deacetylase 7A	Elodie Portales-Casamar	TF Gene
56309	c-myc binding protein	Sarav Sundararajan	Probably Not a TF
56309	c-myc binding protein	Rob Sladek	TF Gene Candidate
65100	zinc finger protein of the cerebellum 5	Gwenael Breard	TF Gene Candidate
65100	zinc finger protein of the cerebellum 5	Wyeth Wasserman	TF Gene
69792	mediator of RNA polymerase II transcription, subunit 6 homolog (yeast)	Sarav Sundararajan	Not a TF
69792	mediator of RNA polymerase II transcription, subunit 6 homolog (yeast)	Andrew Kwon	TF Gene Candidate
71458	Bcl6 interacting corepressor	Shannan HoSui	TF Gene Candidate
71458	Bcl6 interacting corepressor	Andrew Kwon	TF Gene Candidate
71950	Nanog homeobox	Rob Sladek	TF Gene
71950	Nanog homeobox	Tim Hughes	TF Gene
75901	MAD homolog 4 interacting transcription coactivator 1	Sarav Sundararajan	Probably Not a TF
75901	MAD homolog 4 interacting transcription coactivator 1	Rob Sladek	TF Gene Candidate
83796	SWI/SNF related, matrix associated, actin dependent regulator of chromatin, subfamily d, member 2	Debra Fulton	TF Gene Candidate
83796	SWI/SNF related, matrix associated, actin dependent regulator of chromatin, subfamily d, member 2	Wyeth Wasserman	TF Gene Candidate
94222	oligodendrocyte transcription factor 3	Rob Sladek	Indeterminate
94222	oligodendrocyte transcription factor 3	Tim Hughes	TF Gene Candidate
94353	high mobility group nucleosomal binding domain 3	Jared Roach	TF Gene Candidate
94353	high mobility group nucleosomal binding domain 3	Rob Sladek	TF Gene

Table S2. Quality assurance gene annotation pair judgments (continued)

Gene ID	Description	Reviewer	Judgment
110805	forkhead box E1 (thyroid transcription factor 2)	Wyeth Wasserman	TF Gene Candidate
110805	forkhead box E1 (thyroid transcription factor 2)	David Martin	TF Gene Candidate
114142	forkhead box P2	Rob Sladek	TF Gene
114142	forkhead box P2	Tim Hughes	TF Gene
114741	suppressor of Ty 16 homolog (S. cerevisiae)	Jared Roach	Not a TF
114741	suppressor of Ty 16 homolog (S. cerevisiae)	Tim Hughes	TF Gene
209448	homeo box C10	Jared Roach	TF Gene
209448	homeo box C10	Rob Sladek	TF Gene
216285	cartilage homeo protein 1	Rob Sladek	TF Gene
216285	cartilage homeo protein 1	Tim Hughes	TF Gene
223922	activating transcription factor 7	Gwenael Breard	TF Gene
223922	activating transcription factor 7	Sarav Sundararajan	TF Gene
246086	one cut domain, family member 3	David Martin	TF Gene Candidate
246086	one cut domain, family member 3	Alice Chou	TF Gene Candidate

Table S3. Summary of independent annotations of TFs when the same PubMed evidence was used

Gene ID	Gene Symbol	Description	Reviewer	PubMed ID	Judgment	Taxa
18021	Nfatc3	nuclear factor of activated T-cells, cytoplasmic, calcineurin-dependent 3	Sarav Sundararajan	7650004	TF Gene	DNA-Binding: sequence-specific
18021	Nfatc3	nuclear factor of activated T-cells, cytoplasmic, calcineurin-dependent 3	Amy Ticoll	7650004	TF Gene	DNA-Binding: sequence-specific
18021	Nfatc3	nuclear factor of activated T-cells, cytoplasmic, calcineurin-dependent 3	Stuart Lithwick	7650004	TF Gene	DNA-Binding: sequence-specific
15404	Hoxa7	homeo box A7	Sarav Sundararajan	7911971	TF Gene	DNA-Binding: sequence-specific
15407	Hoxb1	homeo box B1	David Martin	7911971	TF Gene	DNA-Binding: sequence-specific
19883	Rora	RAR-related orphan receptor alpha	Jared Roach	7926749	TF Gene	DNA-Binding: sequence-specific
19883	Rora	RAR-related orphan receptor alpha	Sarav Sundararajan	7926749	TF Gene	DNA-Binding: sequence-specific
19883	Rora	RAR-related orphan receptor alpha	Rob Sladek	7926749	TF Gene	DNA-Binding: sequence-specific
14237	Foxd4	forkhead box D4	Amy Ticoll	7957066	TF Gene	DNA-Binding: sequence-specific
14241	Foxl1	forkhead box L1	Jaspar Database	7957066	TF Gene	DNA-Binding: sequence-specific
21815	Tgif1	TG interacting factor 1	Debra Fulton	8537382	TF Gene	DNA-Binding: sequence-specific
245583	Tgif2lx	TGFB-induced factor homeobox 2-like, X-linked	Sarav Sundararajan	8537382	TF Gene	DNA-Binding: sequence-specific
18185	Nrl	neural retina leucine zipper gene	Amy Ticoll	8552602	TF Gene	DNA-Binding: sequence-specific
18185	Nrl	neural retina leucine zipper gene	Stuart Lithwick	8552602	TF Gene	DNA-Binding: non-sequence-specific
18185	Nrl	neural retina leucine zipper gene	Warren Cheung	8552602	TF Gene Candidate	DNA-Binding: sequence-specific; Transcription Factor Binding: TF co-factor binding
83796	Smarcd2	SWI/SNF related, matrix associated, actin dependent regulator of chromatin, subfamily d, member 2	Debra Fulton	8804307	TF Gene Candidate	Transcription Regulatory Activity: heterochromatin interaction/binding
83797	Smarcd1	SWI/SNF related, matrix associated, actin dependent regulator of chromatin, subfamily d, member 1	Tim Hughes	8804307	TF Gene	Transcription Regulatory Activity: heterochromatin interaction/binding

Table S3. Summary of independent annotations of TFs when the same PubMed evidence was used (continued)

Gene ID	Gene Symbol	Description	Reviewer	PubMed ID	Judgment	Taxa
15437	Hoxd8	homeo box D8	Shannan HoSui	8890171	TF Gene Candidate	DNA-Binding: sequence-specific; Transcription Factor Binding: TF co-factor binding
15438	Hoxd9	homeo box D9	Amy Ticoll	8890171	TF Gene	DNA-Binding: sequence-specific
71702	Cdc5l	cell division cycle 5-like (S. pombe)	Sarav Sundararajan	8917598	TF Evidence Conflict	
71702	Cdc5l	cell division cycle 5-like (S. pombe)	Rob Sladek	8917598	TF Evidence Conflict	
11614	Nr0b1	nuclear receptor subfamily 0, group B, member 1	Sarav Sundararajan	9032275	TF Gene	DNA-Binding: non-sequence-specific; Transcription Factor Binding: TF co-factor binding
11614	Nr0b1	nuclear receptor subfamily 0, group B, member 1	Rob Sladek	9032275	TF Gene	DNA-Binding: non-sequence-specific
71702	Cdc5l	cell division cycle 5-like (S. pombe)	Debra Fulton	9038199	TF Evidence Conflict	
71702	Cdc5l	cell division cycle 5-like (S. pombe)	Rob Sladek	9038199	TF Evidence Conflict	
15396	Hoxa11	homeo box A11	Rob Sladek	9079637	TF Gene	DNA-Binding: sequence-specific
15417	Hoxb9	homeo box B9	Andrew Kwon	9079637	TF Gene	DNA-Binding: sequence-specific; Transcription Factor Binding: TF co-factor binding
19668	Rbpjl	recombination signal binding protein for immunoglobulin kappa J region-like	Stuart Lithwick	9111338	TF Gene	DNA-Binding: sequence-specific
19668	Rbpjl	recombination signal binding protein for immunoglobulin kappa J region-like	Warren Cheung	9111338	TF Gene	DNA-Binding: sequence-specific
19668	Rbpjl	recombination signal binding protein for immunoglobulin kappa J region-like	Amy Ticoll	9111338	TF Gene	DNA-Binding: sequence-specific
19668	Rbpjl	recombination signal binding protein for immunoglobulin kappa J region-like	Magdalena Swanson	9111338	TF Gene	DNA-Binding: sequence-specific
17977	Ncoa1	nuclear receptor coactivator 1	Sarav Sundararajan	9192892	TF Gene Candidate	Transcription Factor Binding: TF co-factor binding
17979	Ncoa3	nuclear receptor coactivator 3	Rob Sladek	9192892	TF Gene	Transcription Factor Binding: TF co-factor binding

Table S3. Summary of independent annotations of TFs when the same PubMed evidence was used (continued)

Gene ID	Gene Symbol	Description	Reviewer	PubMed ID	Judgment	Taxa
11614	Nr0b1	nuclear receptor subfamily 0, group B, member 1	Sarav Sundararajan	9384387	TF Gene	DNA-Binding: non-sequence-specific; Transcription Factor Binding: TF co-factor binding
11614	Nr0b1	nuclear receptor subfamily 0, group B, member 1	Debra Fulton	9384387	TF Gene	DNA-Binding: non-sequence-specific; Transcription Factor Binding: TF co-factor binding
11614	Nr0b1	nuclear receptor subfamily 0, group B, member 1	Rob Sladek	9384387	TF Gene	DNA-Binding: non-sequence-specific
13392	Dlx2	distal-less homeobox 2	Andrew Kwon	9415433	TF Gene	DNA-Binding: sequence-specific
13396	Dlx6	distal-less homeobox 6	Andrew Kwon	9415433	TF Gene Candidate	DNA-Binding: sequence-specific
13194	Ddb1	damage specific DNA binding protein 1	Debra Fulton	9418871	TF Gene Candidate	Single stranded RNA/DNA binding; Transcription Factor Binding: TF co-factor binding
107986	Ddb2	damage specific DNA binding protein 2	Sarav Sundararajan	9418871	TF Gene Candidate	Single stranded RNA/DNA binding; Transcription Factor Binding: TF co-factor binding
107986	Ddb2	damage specific DNA binding protein 2	Debra Fulton	9418871	TF Gene Candidate	Single stranded RNA/DNA binding; Transcription Factor Binding: TF co-factor binding
12705	Cited1	Cbp/p300-interacting transactivator with Glu/Asp-rich carboxy-terminal domain 1	Wyeth Wasserman	9434189	TF Gene	Transcription Factor Binding: TF co-factor binding
17684	Cited2	Cbp/p300-interacting transactivator, with Glu/Asp-rich carboxy-terminal domain, 2	Wyeth Wasserman	9434189	TF Gene	Transcription Factor Binding: TF co-factor binding
19727	Rfxank	regulatory factor X-associated ankyrin-containing protein	Tim Hughes	9806546	TF Gene	Transcription Factor Binding: TF co-factor binding
53970	Rfx5	regulatory factor X, 5 (influences HLA class II expression)	Amy Ticoll	9806546	TF Gene Candidate	DNA-Binding: sequence-specific; Transcription Factor Binding: TF co-factor binding
16597	Klf12	Kruppel-like factor 12	Sarav Sundararajan	9858544	TF Gene	DNA-Binding: sequence-specific
16601	Klf9	Kruppel-like factor 9	Debra Fulton	9858544	TF Gene	DNA-Binding: sequence-specific
71702	Cdc5l	cell division cycle 5-like (S. pombe)	Debra Fulton	10570151	TF Evidence Conflict	
71702	Cdc5l	cell division cycle 5-like (S. pombe)	Rob Sladek	10570151	TF Evidence Conflict	

Table S3. Summary of independent annotations of TFs when the same PubMed evidence was used (continued)

Gene ID	Gene Symbol	Description	Reviewer	PubMed ID	Judgment	Taxa
19434	Rax	retina and anterior neural fold homeobox	Amy Ticoll	10625658	TF Gene	DNA-Binding: sequence-specific
19434	Rax	retina and anterior neural fold homeobox	Magdalena Swanson	10625658	TF Gene	DNA-Binding: sequence-specific
19434	Rax	retina and anterior neural fold homeobox	Stuart Lithwick	10625658	TF Gene	DNA-Binding: sequence-specific
19434	Rax	retina and anterior neural fold homeobox	Warren Cheung	10625658	TF Gene	Transcription Factor Binding: TF co-factor binding
11634	Aire	autoimmune regulator (autoimmune polyendocrinopathy candidiasis ectodermal dystrophy)	Sarav Sundararajan	10748110	TF Gene	DNA-Binding: sequence-specific
11634	Aire	autoimmune regulator (autoimmune polyendocrinopathy candidiasis ectodermal dystrophy)	Debra Fulton	10748110	TF Gene	DNA-Binding: non-sequence-specific
11925	Neurog3	neurogenin 3	Amy Ticoll	10757813	TF Gene	DNA-Binding: sequence-specific
11925	Neurog3	neurogenin 3	Magdalena Swanson	10757813	TF Gene	DNA-Binding: sequence-specific
11614	Nr0b1	nuclear receptor subfamily 0, group B, member 1	Sarav Sundararajan	10848616	TF Gene	DNA-Binding: non-sequence-specific; Transcription Factor Binding: TF co-factor binding
11614	Nr0b1	nuclear receptor subfamily 0, group B, member 1	Rob Sladek	10848616	TF Gene	DNA-Binding: non-sequence-specific
56809	Gmeb1	glucocorticoid modulatory element binding protein 1	Andrew Kwon	10894151	TF Gene	DNA-Binding: sequence-specific; Transcription Factor Binding: TF co-factor binding
229004	Gmeb2	glucocorticoid modulatory element binding protein 2	Magdalena Swanson	10894151	TF Gene	DNA-Binding: sequence-specific; Transcription Factor Binding: TF co-factor binding
22772	Zic2	zinc finger protein of the cerebellum 2	Debra Fulton	11053430	TF Gene	DNA-Binding: sequence-specific
22773	Zic3	zinc finger protein of the cerebellum 3	Tim Hughes	11053430	TF Gene	DNA-Binding: sequence-specific
19434	Rax	retina and anterior neural fold homeobox	David Martin	11069920	TF Gene	Transcription Factor Binding: TF co-factor binding
19434	Rax	retina and anterior neural fold homeobox	Warren Cheung	11069920	TF Gene	Transcription Factor Binding: TF co-factor binding
223922	Atf7	activating transcription factor 7	Gwenael Breard	11278933	TF Gene	DNA-Binding: sequence-specific
223922	Atf7	activating transcription factor 7	Sarav Sundararajan	11278933	TF Gene	DNA-Binding: sequence-specific

Table S3. Summary of independent annotations of TFs when the same PubMed evidence was used (continued)

Gene ID	Gene Symbol	Description	Reviewer	PubMed ID	Judgment	Taxa
15417	Hoxb9	homeo box B9	Andrew Kwon	11432851	TF Gene	DNA-Binding: sequence-specific; Transcription Factor Binding: TF co-factor binding
15430	Hoxd10	homeo box D10	David Martin	11432851	TF Gene	DNA-Binding: sequence-specific
20689	Sall3	sal-like 3 (Drosophila)	Magdalena Swanson	11836251	TF Gene Candidate	Transcription Regulatory Activity: heterochromatin interaction/binding
58198	Sall1	sal-like 1 (Drosophila)	Stuart Lithwick	11836251	TF Gene	Transcription Regulatory Activity: heterochromatin interaction/binding
20185	Ncor1	nuclear receptor co-repressor 1	Elodie Portales-Casamar	11997503	TF Gene	Transcription Regulatory Activity: heterochromatin interaction/binding; Transcription Factor Binding: TF co-factor binding
20602	Ncor2	nuclear receptor co-repressor 2	David Martin	11997503	TF Gene Candidate	Transcription Factor Binding: TF co-factor binding
13194	Ddb1	damage specific DNA binding protein 1	Jared Roach	12034848	TF Gene Candidate	Transcription Factor Binding: TF co-factor binding
13194	Ddb1	damage specific DNA binding protein 1	Rob Sladek	12034848	TF Gene Candidate	Single stranded RNA/DNA binding
107986	Ddb2	damage specific DNA binding protein 2	Rob Sladek	12034848	TF Gene Candidate	Transcription Factor Binding: TF co-factor binding
15410	Hoxb3	homeo box B3	Elodie Portales-Casamar	12482716	TF Gene	DNA-Binding: sequence-specific
15412	Hoxb4	homeo box B4	Elodie Portales-Casamar	12482716	TF Gene	DNA-Binding: sequence-specific
18503	Pax1	paired box gene 1	Sarav Sundararajan	12490554	TF Gene	DNA-Binding: sequence-specific
18511	Pax9	paired box gene 9	Rob Sladek	12490554	TF Gene	DNA-Binding: sequence-specific
21415	Tcf3	transcription factor 3	Rob Sladek	14627819	TF Gene	DNA-Binding: sequence-specific
21423	Tcfe2a	transcription factor E2a	Debra Fulton	14627819	TF Gene	DNA-Binding: sequence-specific
74123	Foxp4	forkhead box P4	Amy Ticoll	14701752	TF Gene	DNA-Binding: sequence-specific
114142	Foxp2	forkhead box P2	Rob Sladek	14701752	TF Gene	DNA-Binding: sequence-specific
114142	Foxp2	forkhead box P2	Tim Hughes	14701752	TF Gene	DNA-Binding: sequence-specific

Table S3. Summary of independent annotations of TFs when the same PubMed evidence was used (continued)

Gene ID	Gene Symbol	Description	Reviewer	PubMed ID	Judgment	Taxa
18185	Nrl	neural retina leucine zipper gene	Magdalena Swanson	15001570	TF Gene	DNA-Binding: sequence-specific
18185	Nrl	neural retina leucine zipper gene	Warren Cheung	15001570	TF Gene Candidate	DNA-Binding: sequence-specific; Transcription Factor Binding: TF co-factor binding
20937	Suv39h1	suppressor of variegation 3-9 homolog 1 (Drosophila)	Debra Fulton	15107829	TF Gene	Transcription Factor Binding: TF co-factor binding
64707	Suv39h2	suppressor of variegation 3-9 homolog 2 (Drosophila)	Sarav Sundararajan	15107829	TF Gene Candidate	Transcription Regulatory Activity: heterochromatin interaction / binding; Transcription Factor Binding: TF co-factor binding
18612	Etv4	ets variant gene 4 (E1A enhancer binding protein, E1AF)	Stuart Lithwick	15138262	TF Gene	DNA-Binding: sequence-specific; Transcription Factor Binding: TF co-factor binding
104156	Etv5	ets variant gene 5	Rob Sladek	15138262	TF Gene	DNA-Binding: sequence-specific
93760	Arid1a	AT rich interactive domain 1A (Swi1 like)	Jared Roach	15170388	TF Gene	DNA-Binding: non-sequence-specific
239985	Arid1b	AT rich interactive domain 1B (Swi1 like)	Amy Ticoll	15170388	TF Gene	Transcription Regulatory Activity: heterochromatin interaction / binding; DNA-Binding: non-sequence-specific; Transcription Factor Binding: TF co-factor binding
18021	Nfatc3	nuclear factor of activated T-cells, cytoplasmic, calcineurin-dependent 3	Magdalena Swanson	15173172	TF Gene	DNA-Binding: sequence-specific
18021	Nfatc3	nuclear factor of activated T-cells, cytoplasmic, calcineurin-dependent 3	Stuart Lithwick	15173172	TF Gene	DNA-Binding: sequence-specific
18021	Nfatc3	nuclear factor of activated T-cells, cytoplasmic, calcineurin-dependent 3	Warren Cheung	15173172	TF Gene	DNA-Binding: sequence-specific; Transcription Factor Binding: TF co-factor binding
20671	Sox17	SRY-box containing gene 17	Elodie Portales-Casamar	15220343	TF Gene	DNA-Binding: sequence-specific
20680	Sox7	SRY-box containing gene 7	Amy Ticoll	15220343	TF Gene	DNA-Binding: sequence-specific
19290	Pura	purine rich element binding protein A	Rob Sladek	15282343	TF Gene	Single stranded RNA/DNA binding
19291	Purb	purine rich element binding protein B	Amy Ticoll	15282343	TF Gene	Single stranded RNA/DNA binding

Table S3. Summary of independent annotations of TFs when the same PubMed evidence was used (continued)

Gene ID	Gene Symbol	Description	Reviewer	PubMed ID	Judgment	Taxa
22774	Zic4	zinc finger protein of the cerebellum 4	Gwenael Breard	15465018	TF Gene Candidate	DNA-Binding: sequence-specific
65100	Zic5	zinc finger protein of the cerebellum 5	Wyeth Wasserman	15465018	TF Gene	DNA-Binding: sequence-specific
18612	Etv4	ets variant gene 4 (E1A enhancer binding protein, E1AF)	Stuart Lithwick	15466854	TF Gene	DNA-Binding: sequence-specific; Transcription Factor Binding: TF co-factor binding
104156	Etv5	ets variant gene 5	Rob Sladek	15466854	TF Gene	DNA-Binding: sequence-specific
211323	Nrg1	neuregulin 1	Amy Ticoll	15494726	TF Gene	Transcription Factor Binding: TF co-factor binding
211323	Nrg1	neuregulin 1	Magdalena Swanson	15494726	TF Gene Candidate	Transcription Factor Binding: TF co-factor binding
211323	Nrg1	neuregulin 1	Stuart Lithwick	15494726	TF Gene Candidate	Transcription Factor Binding: TF co-factor binding
211323	Nrg1	neuregulin 1	Warren Cheung	15494726	TF Gene	Transcription Factor Binding: TF co-factor binding
66993	Smarcd3	SWI/SNF related, matrix associated, actin dependent regulator of chromatin, subfamily d, member 3	Alice Chou	15525990	TF Gene Candidate	Transcription Factor Binding: TF co-factor binding
83797	Smarcd1	SWI/SNF related, matrix associated, actin dependent regulator of chromatin, subfamily d, member 1	Tim Hughes	15525990	TF Gene	Transcription Regulatory Activity: heterochromatin interaction /binding
12677	Vsx2	visual system homeobox 2	Elodie Portales-Casamar	15647262	TF Gene	DNA-Binding: sequence-specific
114889	Vsx1	visual system homeobox 1 homolog (zebrafish)	Tim Hughes	15647262	TF Gene	DNA-Binding: sequence-specific
11634	Aire	autoimmune regulator (autoimmune polyendocrinopathy candidiasis ectodermal dystrophy)	Debra Fulton	15649436	TF Gene	DNA-Binding: non-sequence-specific
11634	Aire	autoimmune regulator (autoimmune polyendocrinopathy candidiasis ectodermal dystrophy)	Rob Sladek	15649436	TF Gene	DNA-Binding: non-sequence-specific
107503	Atf5	activating transcription factor 5	Debra Fulton	15735663	TF Gene	DNA-Binding: sequence-specific
223922	Atf7	activating transcription factor 7	Sarav Sundararajan	15735663	TF Gene	DNA-Binding: sequence-specific

Table S3. Summary of independent annotations of TFs when the same PubMed evidence was used (continued)

Gene ID	Gene Symbol	Description	Reviewer	PubMed ID	Judgment	Taxa
15395	Hoxa10	homeo box A10	Jared Roach	15886193	TF Gene	DNA-Binding: sequence-specific
15416	Hoxb8	homeo box B8	Debra Fulton	15886193	TF Gene	DNA-Binding: sequence-specific
15110	Hand1	heart and neural crest derivatives expressed transcript 1	Amy Ticoll	16043483	TF Gene	Transcription Factor Binding: TF co-factor binding
15110	Hand1	heart and neural crest derivatives expressed transcript 1	Stuart Lithwick	16043483	TF Gene	DNA-Binding: sequence-specific
15110	Hand1	heart and neural crest derivatives expressed transcript 1	Warren Cheung	16043483	TF Gene	DNA-Binding: sequence-specific; Transcription Factor Binding: TF co-factor binding
55942	Sertad1	SERTA domain containing 1	Elodie Portales-Casamar	16098148	TF Gene	Transcription Factor Binding: TF co-factor binding
58172	Sertad2	SERTA domain containing 2	Amy Ticoll	16098148	TF Gene	Transcription Factor Binding: TF co-factor binding
27386	Npas3	neuronal PAS domain protein 3	Amy Ticoll	16172381	TF Gene Candidate	DNA-Binding: sequence-specific
27386	Npas3	neuronal PAS domain protein 3	Stuart Lithwick	16172381	TF Gene Candidate	Transcription Regulatory Activity: heterochromatin interaction / binding
27386	Npas3	neuronal PAS domain protein 3	Warren Cheung	16172381	TF Gene Candidate	Transcription Factor Binding: TF co-factor binding
11925	Neurog3	neurogenin 3	Amy Ticoll	16511571	TF Gene	DNA-Binding: sequence-specific
11925	Neurog3	neurogenin 3	Magdalena Swanson	16511571	TF Gene	DNA-Binding: sequence-specific
11925	Neurog3	neurogenin 3	Stuart Lithwick	16511571	TF Gene	DNA-Binding: non-sequence-specific
11925	Neurog3	neurogenin 3	Warren Cheung	16511571	TF Gene	DNA-Binding: sequence-specific
73181	Nfatc4	nuclear factor of activated T-cells, cytoplasmic, calcineurin-dependent 4	Magdalena Swanson	16644691	TF Gene	DNA-Binding: sequence-specific
73181	Nfatc4	nuclear factor of activated T-cells, cytoplasmic, calcineurin-dependent 4	Stuart Lithwick	16644691	TF Gene	DNA-Binding: sequence-specific
73181	Nfatc4	nuclear factor of activated T-cells, cytoplasmic, calcineurin-dependent 4	Warren Cheung	16644691	TF Gene	DNA-Binding: sequence-specific

Table S4. Detected DNA-binding domains predicted in judged TF genes using PFAM and Superfamily group models

PFAM HMM	Superfamily Relationship
AF-4	
ARID	ARID-like
AT_hook	
Beta-trefoil	DNA-binding protein LAG-1 (CSL)
bZIP_1	
bZIP_2	
bZIP_Maf	A DNA-binding domain in eukaryotic transcription factors
CBFB_NFYA	
CBFD_NFYB_HMF	
CP2	
CUT	
DDT	
E2F_TDP	Winged helix DNA-binding domain
Ets	Winged helix DNA-binding domain
Fork_head	Winged helix DNA-binding domain
GATA	Glucocorticoid receptor-like (DNA-binding domain)
GCM	GCM domain
GTF2I	
HLH	HLH, helix-loop-helix DNA-binding domain
HMG_box	HMG-box
Homeobox	Homeodomain-like
HSF_DNA-bind	Winged helix DNA-binding domain
HTH_psq	Homeodomain-like
IRF	Winged helix DNA-binding domain
LAG1-DNAbind	p53-like transcription factors
MBD	DNA-binding domain
MH1	SMAD MH1 domain
Myb_DNA-binding	Homeodomain-like
P53	p53-like transcription factors
PAX	Homeodomain-like
Pou	
RFX_DNA_binding	Winged helix DNA-binding domain
RHD	p53-like transcription factors

Table S4. Detected DNA-binding domains predicted in judged TF genes using PFAM and Superfamily group models (continued)

PFAM HMM	Superfamily Relationship
RNA_pol_Rpb2_1	beta and beta-prime subunits of DNA dependent RNA-polymerase
RNA_pol_Rpb2_2	beta and beta-prime subunits of DNA dependent RNA-polymerase
RNA_pol_Rpb2_3	beta and beta-prime subunits of DNA dependent RNA-polymerase
RNA_pol_Rpb2_4	beta and beta-prime subunits of DNA dependent RNA-polymerase
RNA_pol_Rpb2_5	beta and beta-prime subunits of DNA dependent RNA-polymerase
RNA_pol_Rpb2_6	beta and beta-prime subunits of DNA dependent RNA-polymerase
RNA_pol_Rpb2_7	beta and beta-prime subunits of DNA dependent RNA-polymerase
Runt	p53-like transcription factors
SAND	SAND domain-like
SLIDE	Homeodomain-like
SRF-TF	SRF-like
STAT_bind	p53-like transcription factors
T-box	p53-like transcription factors
TBP	TATA-box binding protein-like
TEA	
TF_AP-2	
TF_Otx	
zf-C2H2	C2H2 and C2HC zinc fingers
zf-C2HC	CCHHC domain
zf-C4	Glucocorticoid receptor-like (DNA-binding domain)
zf-CXXC	

Table S5. DNA-binding classification extensions to Luscombe *et al*. classification system

**Protein Structure Group**	**Protein Structure Family**	**Description**	**Comments**
1.2) Winged Helix-Turn-Helix		The winged HTH motif is an extension of the HTH group, which is characterized by a third or fourth alpha-helix and an adjacent beta-sheet.	Group description modification
1.1) Helix-Turn-Helix	100) Myb Domain Family	The Myb vertebrate DBD consists of three tandem repeats of 51 to 53 amino acid residues from the amino acid terminus, herein referred to as R1, R2, and R3 (Kanei-Ishii et al. 1990). Each repeat contains three helices of a helix-turn-helix motif α1, α2, and α3 (Ogata et al. 1992; Ogata et al. 1995) with R2 and R3 involved in specific DNA recognition and R1 covers the DNA position next to the R2 binding site. The R1, R2, and R3 bind to DNA mainly in the major groove (Tahirov et al. 2002).<br>The relationship between the protein domains of Myb and Rap1 is heavily covered in the literature. While there is a clear sequence similarity, there are important differences between the two regions in the proteins. A study by Hanaoka et al. (Hanaoka et al. 2001) compares the Myb-like domains. The human Rap1 protein fragment solution structure PDB: 1FEX appears to share structural similarity to Myb. However, yeast Rap1 associated with DNA (available in PDB 1IGN) does not align well with Myb. It is noteworthy that the yeast (SC) protein does have observed DNA binding capacity while the human seems to require interaction with TRF2 for association with DNA (O'Connor et al. 2004).	Family added
1.2) Winged Helix-Turn-Helix	101) GTF2I Domain Family	DNA-binding studies suggest that GTF2I domain binds DNA (Rauhala et al. 2005); (Vullhorst et al. 2003). At the time of our analysis, no DNA-bound protein structures were available for review. However, structural alignments using an NMR-based structure suggest that this domain may take on a Helix-Turn-Helix configuration. Based on its unique conformation we predict that its DNA binding mechanism will likely support its own HTH family.	Family added – predicted

Table S5. DNA-binding classification extensions (continued)

**Protein Structure Group**	**Protein Structure Family**	**Description**	**Comments**
1.2) Winged Helix-Turn-Helix	102) Forkhead Domain Family	The forkhead domain binds DNA as a monomer (Clark et al. 1993). Three or four helices are set against a small three-stranded antiparallel beta-sheet from which two large loops extend (Clark et al. 1993; van Dongen et al. 2000). DNA binding occurs largely through the third helix inserted in the major groove of the DNA (Clark et al. 1993). The wings of the forkhead domain also make contact with the DNA and may contribute to sequence specificity (Bravieri et al. 1997).	Family added
1.2) Winged Helix-Turn-Helix	103) RFX Domain Family	The hRFX1 DBD consists of three alpha-helices, three beta-strands, and three connecting loops (Gajiwala et al. 2000). The third loop, connecting beta-strands S2 and S3, forms wing W1 of the winged-helix motif and makes contact with the DNA in the major groove, along with beta-strands S2 and S3. In contrast to other winged-helix DBDs, RFX has only 1 wing.	Family added
2) Zinc-coordinating Group	104) GATA Domain Family	The GATA domain is composed of a core: a zinc coordinated by four cysteines and a carboxyl-terminal tail (Omichinski et al. 1993). Specifically, the core consists of a two anti-parallel Beta-sheets and an alpha helix connected to a long loop that tethers a carboxyl-terminal tail. DNA contact is made through a helix and loop connecting the two Beta-sheets in the major groove and carboxyl-terminal tail around the DNA making contact with the minor groove (Omichinski et al. 1993).	Family added
2) Zinc-coordinating Group	105) Glial Cells Missing (GCM) Domain Family	The GCM domain is composed of one five- and one three-stranded Beta sheet, with three small helical segments packed against the same side of the two beta-sheets (Cohen et al. 2003). The 5-stranded Beta-sheet is inserted into the major groove of the DNA. Residues from the edge of the Beta sheet and the following loop and strand contact the DNA backbone and bases - providing the sequence specificity (Cohen et al. 2003).	Family added

Table S5. DNA-binding classification extensions (continued)

Protein Structure Group	Protein Structure Family	Description	Comments
2) Zinc-coordinating Group	106) MH1 Domain Family	Smad3 MH1 domain consists of four alpha-helices, six beta-strands, and five loops. The Smad MH1 domain contains a novel DNA-binding motif, an 11-residue beta-hairpin (formed by the second and third beta-strands), is embedded asymmetrically in the major groove of DNA (Chai et al. 2003). The MH1 domain contains a bound zinc atom coordinated by three cysteines and one histidine. Removal of the zinc atom results in reduced DNA binding activity. However, not all MH1-containing proteins can bind to DNA (such as Smad1). Sequence analyses suggest that the DNA-binding domains of CTF/NFI and SMAD MH1 demonstrate significant sequence homology (Stefancsik et al. 2003).	Family added
4) Other Alpha-Helix Group	107) Sand Domain Family	The GMEb1 Sand domain adopts a compact fold with an alpha-helical face and a twisted Beta-sheet face (Surdo et al. 2003). At the time of our analysis, no DNA-bound protein structures were available for review. However, the DNA binding surface has been mapped to the alpha-helical region encompassing the KDWK motif (Bottomley et al. 2001; Surdo et al. 2003). The GMEB1 SAND domain contains a zinc-binding motif and, although the zinc ion is not required for DNA binding, it plays a role in determining the C-terminal conformation of the GMEB1 SAND domain (Surdo et al. 2003).	Family added – predicted
6) Beta Hairpin_Ribbon Group	108) Methyl-CpG-binding Domain, Family (MBD)	The MBD folds into an alpha/beta sandwich structure, which is comprised of a layer of twisted beta-sheet, backed by another layer formed by the alpha helix 1 and a hairpin loop at the C terminus. The beta sheet is composed of two long inner strands (beta-strand 2 and beta-strand 3) sandwiched by two shorter outer strands (beta-strand 1 and beta-strand 4) (Ohki et al. 2001). A section of the inner strands that projects beyond the outer strands is embedded in the major groove at the target DNA site and, together with the loop that links beta-strand 2 and beta-strand 3, forms the principal DNA interface. The twisted beta-sheet is angled within the major groove to wedge the C-terminal part of beta-strand 4. This orientation of the beta-sheet allows alpha-helix 1 and loop 2 to mediate major groove contacts with DNA (Ohki et al. 2001).	Family added

Table S5. DNA-binding classification extensions (continued)

Protein Structure Group	Protein Structure Family	Description	Comments
1.2) Winged Helix	109) Arid Domain Family	Arid family proteins can be grouped into subfamilies based on sequence similarity. A majority of these subfamilies bind DNA without obvious sequence specificity (Patsialou et al. 2005)). Arid appears to interact with both the major and minor DNA grooves. Major groove DNA contact is made through insertion of a loop (Kim et al. 2004) and/or an α-helix (Iwahara et al. 2002).	Family added
7) Other	110) Runt Domain Family	The Runt domain recognizes specific bases in both the major and minor grooves of the DNA, and binding is accomplished mainly using loops. CBFα Run domain makes contact with the DNA consensus sequence using three loop-containing regions: Beta-strand 3- Loop 3, Beta-strand 9 – Loop 9, and Beta-strand 12 – Loop 12 (Backstrom et al. 2002). The first and third loop interact with the major groove, while the second interacts with the minor groove. Two chloride ions bind to the Runt domain one of which is situated at the DNA-binding surface and are shown to have a positive effect on DNA binding (Backstrom et al. 2002).<br>Structural comparisons demonstrate that the s-type Ig fold found in the Runt domain is conserved in the Ig folds found in the DNA-binding domains of NF-kappaB, NFAT, p53, STAT-1, and the T-domain. The differences among these proteins arise in the connecting loop regions where short additional secondary structural elements have been added that in some cases interact with the core Ig scaffold (Berardi et al. 1999). These proteins appear to form a family of structurally and functionally related DNA-binding domains. Unlike the other members of this family, the Runt domain utilizes loops at both ends of the Ig fold for DNA recognition (Berardi et al. 1999).	Added family

Table S5. DNA-binding classification extensions (continued)

Protein Structure Group	Protein Structure Family	Description	Comments
1.2) Winged Helix-Turn-Helix	111) Slide Domain Family	The three core helices of the Slide domain superimpose well with c-Myb repeats. The slide domain appears to be highly compatible with a role in DNA binding given that it has an overall positive charge and c-MYB DNA-contacting residues are largely conserved in the slide domain (Grune et al. 2003). At the time of our analysis, no DNA-bound protein structures were available for review. However, given its similarities with the c-Myb protein structure, we predict that the Slide DNA binding structure may take on a helix-turn-helix (HTH) conformation and, as such, have classified in its own HTH family and will revisit this assignment when a DNA-bound structure is available.	Family added – predicted
7) Other	112) Beta-trefoil-like	The beta-trefoil domain (BTD) is a capped beta-barrel. The prototypical BTD consists of four strands repeated in a three-fold arrangement, where beta-strands 1 and 4 form the walls of the barrel and beta – strands 1 and 2 form the cap of the barrel. However, the CSL protein's BTD posses minor deviations from this (Kovall et al. 2004). In CSL the BTD makes specific contact with the DNA minor groove DNA and non-specific contact with the phosphate-ribose backbone (Kovall et al. 2004).	Family added
7)Other	113) DNA-Binding LAG-1-like	Lag-1 DNA binding domain is composed of a seven-stranded beta barrel organized into a sandwich composed of three- and four-stranded beta sheets (characteristics of an IG-like fold) (Kovall et al. 2004). In the CSL protein, the Lag-1 DNA binding domain interacts with the major groove of the DNA (Kovall et al. 2004).	Family added
2) Zinc-coordinating Group	114) Non-methyl CpG-binding CXXC Domain	Three cysteine residues in two CGXCXXC motifs provide coordination for two zinc ions. Both motifs adopt a similar conformation in which the second and third cysteines are contained within a small helix or form a small helix-turn-helix. At the time of our analysis, no DNA-bound protein structures were available for review. However, NMR binding and mutagenesis data define the CXXC domain as the non-methyl CpG DNA binding interface (Allen et al. 2006).	Family added – predicted

Table S5. DNA-binding classification extensions (continued)

Protein Structure Group	Protein Structure Family	Description	Comments
4) Other Alpha Helix Group	115) NF-Y CCAAT-Binding Protein Family	CBF/NF-Y is a heterotrimeric complex composed of NF-YA, NF-YB and NF-YC, which are all are required for DNA binding(Romier et al. 2003). CBF NF-YC and NF-YB are homologous to histones H2A and H2B (Sinha et al. 1995). Although there are no DNA-bound structures available for review, DNA recognition appears to involve both minor and major grooves interactions (inferred by methylation interference patterns) (Ronchi et al. 1995).	
999) Unclassified Structure	901) CP2 Transcription Factor Domain Family	The DNA binding domain of CP2-like genes has been experimentally identified (Rodda et al. 2001) and appears to be somewhat conserved in the fly Grainyhead TF (Uv et al. 1994). At the time of our analysis, no protein structure was available for review.	Unclassified DBD structure
999) Unclassified Structure	902) AF-4 Protein Family	AF-4 proteins have been shown to bind DNA *in vitro* (Ma et al. 1996). However, the structural DNA binding mechanism is unclear.	Unclassified DBD structure
999) Unclassified Structure	903) DNA binding homeobox and Different Transcription factors (DDT)	Experimental analysis of Fac1 (a truncated version of bromodomain PHD finger transcription factors) demonstrates that the N-terminal region, which includes the DDT domain, is involved in DNA-binding (Jordan-Sciutto et al. 1999). Secondary structure predictions suggest that DDT is composed of 3 alpha helices. However, fold recognition comparisons do not suggest any significant similarity to known DNA-RNA binding alpha-helical bundles (Doerks et al. 2001).	Unclassified DBD structure
999) Unclassified Structure	904) AT-hook Domain Family	The AT-Hook domain is the DNA-binding domain of the HMGI(Y) family of proteins. At the time of our analysis, the only DNA-bound structure available was HMGA1 (HMG-I(Y)). Unfortunately this structure is derived from NMR data produced over a decade ago and does not appear sufficiently detailed to confidently assess a structural family.	Unclassified DBD structure

Table S5. DNA-binding classification extensions (continued)

**Protein Structure Group**	**Protein Structure Family**	**Description**	**Comments**
999) Unclassified Structure	905) Nuclear Factor I - CCAAT-binding Transcription Factor (NFI-CTF) Family	Nuclear Factor I NFI/CTI TFs can form both homo- and heterodimers (Kruse et al. 1994). At the time of our analysis, no protein structure was available for review. The family contains a conserved N-terminal DNA-binding domain (within the first 240 amino acids of CTF/NFI) (Gournari et al. 1990). Chicken and mammalian homolog genes incorporating this domain are NFI-A, NFI-B, and NFI-C (Rupp et al. 1990). Sequence analyses suggest that the DNA-binding domains of CTF/NFI and SMAD MH1 demonstrate significant sequence homology (Stefancsik et al. 2003).	Unclassified DBD structure

References for Table S5

Allen, M.D., C.G. Grummitt, C. Hilcenko, S.Y. Min, L.M. Tonkin, C.M. Johnson, S.M. Freund, M. Bycroft, and A.J. Warren. 2006. Solution structure of the nonmethyl-CpG-binding CXXC domain of the leukaemia-associated MLL histone methyltransferase. *Embo J* **25:** 4503-4512.

Backstrom, S., M. Wolf-Watz, C. Grundstrom, T. Hard, T. Grundstrom, and U.H. Sauer. 2002. The RUNX1 Runt domain at 1.25A resolution: a structural switch and specifically bound chloride ions modulate DNA binding. *J Mol Biol* **322:** 259-272.

Berardi, M.J., C. Sun, M. Zehr, F. Abildgaard, J. Peng, N.A. Speck, and J.H. Bushweller. 1999. The Ig fold of the core binding factor alpha Runt domain is a member of a family of structurally and functionally related Ig-fold DNA-binding domains. *Structure* **7:** 1247-1256.

Bottomley, M.J., M.W. Collard, J.I. Huggenvik, Z. Liu, T.J. Gibson, and M. Sattler. 2001. The SAND domain structure defines a novel DNA-binding fold in transcriptional regulation. *Nat Struct Biol* **8:** 626-633.

Bravieri, R., T. Shiyanova, T.H. Chen, D. Overdier, and X. Liao. 1997. Different DNA contact schemes are used by two winged helix proteins to recognize a DNA binding sequence. *Nucleic Acids Res* **25:** 2888-2896.

Chai, J., J.W. Wu, N. Yan, J. Massague, N.P. Pavletich, and Y. Shi. 2003. Features of a Smad3 MH1-DNA complex. Roles of water and zinc in DNA binding. *J Biol Chem* **278:** 20327-20331.

Clark, K.L., E.D. Halay, E. Lai, and S.K. Burley. 1993. Co-crystal structure of the HNF-3/fork head DNA-recognition motif resembles histone H5. *Nature* **364:** 412-420.

Cohen, S.X., M. Moulin, S. Hashemolhosseini, K. Kilian, M. Wegner, and C.W. Muller. 2003. Structure of the GCM domain-DNA complex: a DNA-binding domain with a novel fold and mode of target site recognition. *Embo J* **22:** 1835-1845.

Doerks, T., R. Copley, and P. Bork. 2001. DDT -- a novel domain in different transcription and chromosome remodeling factors. *Trends Biochem Sci* **26:** 145-146.

Gajiwala, K.S., H. Chen, F. Cornille, B.P. Roques, W. Reith, B. Mach, and S.K. Burley. 2000. Structure of the winged-helix protein hRFX1 reveals a new mode of DNA binding. *Nature* **403:** 916-921.

Gounari, F., R. De Francesco, J. Schmitt, P. van der Vliet, R. Cortese, and H. Stunnenberg. 1990. Amino-terminal domain of NF1 binds to DNA as a dimer and activates adenovirus DNA replication. *Embo J* **9:** 559-566.

Grune, T., J. Brzeski, A. Eberharter, C.R. Clapier, D.F. Corona, P.B. Becker, and C.W. Muller. 2003. Crystal structure and functional analysis of a nucleosome recognition module of the remodeling factor ISWI. *Mol Cell* **12:** 449-460.

Hanaoka, S., A. Nagadoi, S. Yoshimura, S. Aimoto, B. Li, T. de Lange, and Y. Nishimura. 2001. NMR structure of the hRap1 Myb motif reveals a canonical three-helix bundle lacking the positive surface charge typical of Myb DNA-binding domains. *J Mol Biol* **312:** 167-175.

Iwahara, J., M. Iwahara, G.W. Daughdrill, J. Ford, and R.T. Clubb. 2002. The structure of the Dead ringer-DNA complex reveals how AT-rich interaction domains (ARIDs) recognize DNA. *Embo J* **21:** 1197-1209.

Jordan-Sciutto, K.L., J.M. Dragich, and R. Bowser. 1999. DNA binding activity of the fetal Alz-50 clone 1 (FAC1) protein is enhanced by phosphorylation. *Biochem Biophys Res Commun* **260:** 785-789.

Kanei-Ishii, C., A. Sarai, T. Sawazaki, H. Nakagoshi, D.N. He, K. Ogata, Y. Nishimura, and S. Ishii. 1990. The tryptophan cluster: a hypothetical structure of the DNA-binding domain of the myb protooncogene product. *J Biol Chem* **265:** 19990-19995.

Kim, S., Z. Zhang, S. Upchurch, N. Isern, and Y. Chen. 2004. Structure and DNA-binding sites of the SWI1 AT-rich interaction domain (ARID) suggest determinants for sequence-specific DNA recognition. *J Biol Chem* **279:** 16670-16676.

Kovall, R.A. and W.A. Hendrickson. 2004. Crystal structure of the nuclear effector of Notch signaling, CSL, bound to DNA. *Embo J* **23:** 3441-3451.

Kruse, U. and A.E. Sippel. 1994. Transcription factor nuclear factor I proteins form stable homo- and heterodimers. *FEBS Lett* **348:** 46-50.

Ma, C. and L.M. Staudt. 1996. LAF-4 encodes a lymphoid nuclear protein with transactivation potential that is homologous to AF-4, the gene fused to MLL in t(4;11) leukemias. *Blood* **87:** 734-745.

O'Connor, M.S., A. Safari, D. Liu, J. Qin, and Z. Songyang. 2004. The human Rap1 protein complex and modulation of telomere length. *J Biol Chem* **279:** 28585-28591.

Ogata, K., H. Hojo, S. Aimoto, T. Nakai, H. Nakamura, A. Sarai, S. Ishii, and Y. Nishimura. 1992. Solution structure of a DNA-binding unit of Myb: a helix-turn-helix-related motif with conserved tryptophans forming a hydrophobic core. *Proc Natl Acad Sci U S A* **89:** 6428-6432.

Ogata, K., S. Morikawa, H. Nakamura, H. Hojo, S. Yoshimura, R. Zhang, S. Aimoto, Y. Ametani, Z. Hirata, A. Sarai et al. 1995. Comparison of the free and DNA-complexed forms of the DNA-binding domain from c-Myb. *Nat Struct Biol* **2:** 309-320.

Ohki, I., N. Shimotake, N. Fujita, J. Jee, T. Ikegami, M. Nakao, and M. Shirakawa. 2001. Solution structure of the methyl-CpG binding domain of human MBD1 in complex with methylated DNA. *Cell* **105:** 487-497.

Omichinski, J.G., G.M. Clore, O. Schaad, G. Felsenfeld, C. Trainor, E. Appella, S.J. Stahl, and A.M. Gronenborn. 1993. NMR structure of a specific DNA complex of Zn-containing DNA binding domain of GATA-1. *Science* **261:** 438-446.

Patsialou, A., D. Wilsker, and E. Moran. 2005. DNA-binding properties of ARID family proteins. *Nucleic Acids Res* **33:** 66-80.

Rodda, S., S. Sharma, M. Scherer, G. Chapman, and P. Rathjen. 2001. CRTR-1, a developmentally regulated transcriptional repressor related to the CP2 family of transcription factors. *J Biol Chem* **276:** 3324-3332.

Romier, C., F. Cocchiarella, R. Mantovani, and D. Moras. 2003. The NF-YB/NF-YC structure gives insight into DNA binding and transcription regulation by CCAAT factor NF-Y. *J Biol Chem* **278:** 1336-1345.

Ronchi, A., M. Bellorini, N. Mongelli, and R. Mantovani. 1995. CCAAT-box binding protein NF-Y (CBF, CP1) recognizes the minor groove and distorts DNA. *Nucleic Acids Res* **23:** 4565-4572.

Rupp, R. A., Kruse, U., Multhaup, G., Gobel, U., Beyreuther, K., Sippel, A. E. 1990. Chicken NFI/TGGCA proteins are encoded by at least three independent genes: NFI-A, NFI-B and NFI-C with homologues in mammalian genomes. *Nucleic Acids Res* **18:** 2607-2616

Sinha, S., S.N. Maity, J. Lu, and B. de Crombrugghe. 1995. Recombinant rat CBF-C, the third subunit of CBF/NFY, allows formation of a protein-DNA complex with CBF-A and CBF-B and with yeast HAP2 and HAP3. *Proc Natl Acad Sci U S A* **92:** 1624-1628.

Stefancsik, R. and S. Sarkar. 2003. Relationship between the DNA binding domains of SMAD and NFI/CTF transcription factors defines a new superfamily of genes. *DNA Seq* **14:** 233-239.

Surdo, P.L., M.J. Bottomley, M. Sattler, and K. Scheffzek. 2003. Crystal structure and nuclear magnetic resonance analyses of the SAND domain from glucocorticoid modulatory element binding protein-1 reveals deoxyribonucleic acid and zinc binding regions. *Mol Endocrinol* **17:** 1283-1295.

Tahirov, T.H., K. Sato, E. Ichikawa-Iwata, M. Sasaki, T. Inoue-Bungo, M. Shiina, K. Kimura, S. Takata, A. Fujikawa, H. Morii et al. 2002. Mechanism of c-Myb-C/EBP beta cooperation from separated sites on a promoter. *Cell* **108:** 57-70.

Uv, A.E., C.R. Thompson, and S.J. Bray. 1994. The Drosophila tissue-specific factor Grainyhead contains novel DNA-binding and dimerization domains which are conserved in the human protein CP2. *Mol Cell Biol* **14:** 4020-4031.

van Dongen, M.J., A. Cederberg, P. Carlsson, S. Enerback, and M. Wikstrom. 2000. Solution structure and dynamics of the DNA-binding domain of the adipocyte-transcription factor FREAC-11. *J Mol Biol* **296:** 351-359.

Vullhorst, D. and A. Buonanno. 2003. Characterization of general transcription factor 3, a transcription factor involved in slow muscle-specific gene expression. *J Biol Chem* **278:** 8370-8379.

Vullhorst, D. and A. Buonanno. 2005. Multiple GTF2I-like repeats of general transcription factor 3 exhibit DNA binding properties. Evidence for a common origin as a sequence-specific DNA interaction module. *J Biol Chem* **280:** 31722-31731.

Table S6. Protein class counts of genes predicted to contain multiple instances of the same DNA-Binding domain

Protein Group ID	Protein Group Description	Protein Family ID	Protein Family Description	# Genes With Multiple Predicted Instances
1.1	Helix-Turn-Helix Group	100	Myb Domain Family	5
1.1	Helix-Turn-Helix Group	2	Homeodomain Family	22
1.2	Winged Helix-Turn-Helix	101	GTF2I Domain Family	2
1.2	Winged Helix-Turn-Helix	15	Transcription Factor Family	1
2.1	Zinc-coordinating Group	104	GATA Domain Family	5
2.1	Zinc-coordinating Group	114	Non Methyl-CpG-binding CXXC Domain	1
2.1	Zinc-coordinating Group	17	BetaBetaAlpha-zinc finger Family	79
3	Zipper-Type Group	21	Leucine Zipper Family	23
4	Other Alpha-Helix Group	28	High Mobility Group (Box)	3
5	Beta-sheet Group	30	TATA box-binding Family	1
8	Enzyme Group	47	DNA Polymerase-Beta Family	1
999	Unclassified Structure	904	AT-hook Domain Family	2

Table S7. DNA-binding transcription factors predicted to contain two different DNA-binding classes

Gene ID	Gene Symbol	Gene Description	Predicted Protein Group	Predicted Protein Family
11909	*Atf2*	activating transcription factor 2	Zinc-coordinating Group	BetaBetaAlpha-zinc finger Family
11909	*Atf2*	activating transcription factor 2	Zipper-Type Group	Leucine Zipper Family
17190	*Mbd1*	methyl-CpG binding domain protein 1	Zinc-coordinating Group	Non Methyl-CpG-binding CXXC Domain
17190	*Mbd1*	methyl-CpG binding domain protein 1	Beta-Hairpin-Ribbon Group	Methyl-CpG-binding domain, MBD Family
19664	*Rbpj*	recombination signal binding protein for immunoglobuli n kappa	Other	Beta_Trefoil-like Domain Family
19664	*Rbpj*	recombination signal binding protein for immunoglobuli n kappa J region	Other	DNA-binding LAG-1-like Domain Family
19668	*Rbpjl*	recombination signal binding protein for immunoglobuli n kappa J region-like	Other	Beta_Trefoil-like Domain Family
19668	*Rbpjl*	recombination signal binding protein for immunoglobuli n kappa J region-like	Other	DNA-binding LAG-1-like Domain Family
56218	*Patz1*	POZ (BTB) and AT hook containing zinc finger 1	Zinc-coordinating Group	BetaBetaAlpha-zinc finger Family
56218	*Patz1*	POZ (BTB) and AT hook containing zinc finger 1	Unclassified Structure	AT-hook Domain Family

Table S7. DNA-binding transcription factors predicted to contain two different DNA-binding classes (continued)

Gene ID	Gene Symbol	Gene Description	Predicted Protein Group	Predicted Protein Family
116870	*Mta1*	metastasis associated 1	Helix-Turn-Helix Group	Myb Domain Family
116870	*Mta1*	metastasis associated 1	Zinc-coordinating Group	GATA Domain Family
223922	*Atf7*	activating transcription factor 7	Zinc-coordinating Group	BetaBetaAlpha-zinc finger Family
223922	*Atf7*	activating transcription factor 7	Zipper-Type Group	Leucine Zipper Family
214162	*Mll1*	myeloid/lymphoid or mixed-lineage leukemia 1	Zinc-coordinating Group	Non Methyl-CpG-binding CXXC Domain
214162	*Mll1*	myeloid/lymphoid or mixed-lineage leukemia 1	Unclassified Structure	AT-hook Domain Family

Table S8. DNA-binding transcription factors with no detected DNA-binding domains

Gene ID	Gene Symbol	Gene Description
106389	*Eaf2*	ELL associated factor 2
232906	*Grlf1*	glucocorticoid receptor DNA binding factor 1
56461	*Kcnip3*	calsenilin, presenilin binding protein, EF hand transcription factor
104338	*Mynf1*	myeloid nuclear factor 1
11614	*Nr0b1*	nuclear receptor subfamily 0, group B, member 1
74451	*Pgs1*	phosphatidylglycerophosphate synthase 1
50907	*Preb*	prolactin regulatory element binding
79401	*Spz1*	spermatogenic Zip 1
21411	*Tcf20*	transcription factor 20
57432	*Zc3h8*	zinc finger CCCH type containing 8

Table S9. DNA-binding transcription factors with no detected Protein Family class

Gene ID	Gene Symbol	Gene Description	Predicted Protein Group
11545	*Parp1*	poly (ADP-ribose) polymerase family, member 1	Zinc-coordinating Group
14056	*Ezh2*	enhancer of zeste homolog 2 (Drosophila)	Helix-Turn-Helix Group
21804	*Tgfb1i1*	transforming growth factor beta 1 induced transcript 1	Zinc-coordinating Group
245583	*Tgif2lx*	TGFB-induced factor homeobox 2-like, X-linked	Helix-Turn-Helix Group

Table S10. Single-stranded DNA-binding transcription factors

Gene ID	Gene Symbol	Description
245000	*Atr*	ataxia telangiectasia and rad3 related
13194	*Ddb1*	damage specific DNA binding protein 1
107986	*Ddb2*	damage specific DNA binding protein 2
15384	*Hnrpab*	heterogeneous nuclear ribonucleoprotein A/B
50926	*Hnrpdl*	heterogeneous nuclear ribonucleoprotein D-like
15387	*Hnrpk*	heterogeneous nuclear ribonucleoprotein K
17876	*Myef2*	myelin basic protein expression factor 2, repressor
74164	*Nfx1*	nuclear transcription factor, X-box binding 1
18148	*Npm1*	nucleophosmin 1
23983	*Pcbp1*	poly(rC) binding protein 1
18521	*Pcbp2*	poly(rC) binding protein 2
19290	*Pura*	purine rich element binding protein A
19291	*Purb*	purine rich element binding protein B
56381	*Spen*	SPEN homolog, transcriptional regulator (Drosophila)
106021	*Topors*	topoisomerase I binding, arginine/serine-rich
22608	*Ybx1*	Y box protein 1

Table S11. Summary of curated TFCat TFs in Gene Ontology (GO)

GO Annotation Type	Molecular Function Sub-Tree
Excluding GO annotations types:<br>IEA: Inferred from Electronic Annotations<br>ISS: Inferred from Sequence or Structural Similarity Annotations<br>RCA: Inferred from Reviewed Computational Analysis	343 / 882
All GO annotation types (including IEA, ISS, RCA)	409 / 882

Table S12. Comparison summary of TFCat classified HMM DNA-binding domains with the DBD database (DBDdb) resource

	Found in DBD	Not found in DBD
Classified in TFCat	116	16
Not classified in TFCat	68	

Table S13. Superfamily DNA binding domain list comparison with DBD Database (DBDdb) resource

Model ID	Domain Name	In DBD	In TFCat
39848	A DNA-binding domain in eukaryotic transcription factors	Y	Y
43644	A DNA-binding domain in eukaryotic transcription factors	Y	Y
35817	ARID-like		Y
43437	ARID-like		Y
34823	C2H2 and C2HC zinc fingers	Y	Y
34824	C2H2 and C2HC zinc fingers	Y	
34825	C2H2 and C2HC zinc fingers	Y	Y
34826	C2H2 and C2HC zinc fingers	Y	Y
35441	C2H2 and C2HC zinc fingers	Y	Y
35556	C2H2 and C2HC zinc fingers	Y	Y
37351	C2H2 and C2HC zinc fingers	Y	Y
37782	C2H2 and C2HC zinc fingers	Y	Y
40545	C2H2 and C2HC zinc fingers	Y	Y
41311	C2H2 and C2HC zinc fingers	Y	Y
41429	C2H2 and C2HC zinc fingers	Y	Y
42182	C2H2 and C2HC zinc fingers	Y	Y
42220	C2H2 and C2HC zinc fingers	Y	
43688	C2H2 and C2HC zinc fingers	Y	
43689	C2H2 and C2HC zinc fingers	Y	
43730	C2H2 and C2HC zinc fingers	Y	Y
43976	C2H2 and C2HC zinc fingers	Y	Y
43977	C2H2 and C2HC zinc fingers	Y	
43978	C2H2 and C2HC zinc fingers	Y	Y
43982	C2H2 and C2HC zinc fingers	Y	
43983	C2H2 and C2HC zinc fingers	Y	
43984	C2H2 and C2HC zinc fingers	Y	
44259	C2H2 and C2HC zinc fingers	Y	
44260	C2H2 and C2HC zinc fingers	Y	
44261	C2H2 and C2HC zinc fingers	Y	Y
45110	C2H2 and C2HC zinc fingers	Y	Y
45118	C2H2 and C2HC zinc fingers	Y	
45151	C2H2 and C2HC zinc fingers	Y	
45152	C2H2 and C2HC zinc fingers	Y	Y
45249	C2H2 and C2HC zinc fingers	Y	Y
45250	C2H2 and C2HC zinc fingers	Y	
45293	C2H2 and C2HC zinc fingers	Y	Y
45294	C2H2 and C2HC zinc fingers	Y	
45295	C2H2 and C2HC zinc fingers	Y	
45296	C2H2 and C2HC zinc fingers	Y	
45297	C2H2 and C2HC zinc fingers	Y	Y
45613	C2H2 and C2HC zinc fingers	Y	
45631	C2H2 and C2HC zinc fingers	Y	Y
42508	CCHHC domain	Y	Y
40609	Cysteine-rich DNA binding domain, (DM domain)	Y	

Table S13. Superfamily DNA binding domain list (continued)

Model ID	Domain Name	In DBD	In TFCat
36316	DNA-binding domain	Y	Y
42826	DNA-binding domain	Y	
41800	GCM domain	Y	Y
36002	Glucocorticoid receptor-like (DNA-binding domain)	Y	Y
36583	Glucocorticoid receptor-like (DNA-binding domain)	Y	
40006	Glucocorticoid receptor-like (DNA-binding domain)	Y	
40440	Glucocorticoid receptor-like (DNA-binding domain)	Y	
40589	Glucocorticoid receptor-like (DNA-binding domain) Y	Y	
45290	Glucocorticoid receptor-like (DNA-binding domain)	Y	Y
45386	Glucocorticoid receptor-like (DNA-binding domain)	Y	Y
45592	Glucocorticoid receptor-like (DNA-binding domain)	Y	Y
34803	HLH, helix-loop-helix DNA-binding domain	Y	Y
35101	HLH, helix-loop-helix DNA-binding domain	Y	Y
35112	HLH, helix-loop-helix DNA-binding domain	Y	Y
35113	HLH, helix-loop-helix DNA-binding domain	Y	Y
38629	HLH, helix-loop-helix DNA-binding domain	Y	Y
40898	HLH, helix-loop-helix DNA-binding domain	Y	Y
41437	HLH, helix-loop-helix DNA-binding domain	Y	Y
41452	HLH, helix-loop-helix DNA-binding domain	Y	Y
43065	HLH, helix-loop-helix DNA-binding domain	Y	Y
34886	Homeodomain-like	Y	Y
34887	Homeodomain-like	Y	
35079	Homeodomain-like	Y	Y
35379	Homeodomain-like	Y	Y
35402	Homeodomain-like	Y	Y
35403	Homeodomain-like	Y	
35741	Homeodomain-like	Y	
36604	Homeodomain-like	Y	
37777	Homeodomain-like	Y	
37778	Homeodomain-like	Y	Y
38474	Homeodomain-like	Y	
38986	Homeodomain-like	Y	Y
40485	Homeodomain-like	Y	Y
40874	Homeodomain-like	Y	Y
40945	Homeodomain-like	Y	
41016	Homeodomain-like	Y	Y
42267	Homeodomain-like	Y	Y
42468	Homeodomain-like	Y	Y
44368	Homeodomain-like	Y	Y
44899	Homeodomain-like	Y	
45311	Homeodomain-like	Y	
45348	Homeodomain-like	Y	Y
45634	Homeodomain-like	Y	

Table S13. Superfamily DNA binding domain list (continued)

Model ID	Domain Name	In DBD	In TFCat
35201	lambda repressor-like DNA-binding domains	Y	Y
36752	lambda repressor-like DNA-binding domains	Y	Y
38966	lambda repressor-like DNA-binding domains	Y	Y
43707	lambda repressor-like DNA-binding domains	Y	
37961	Nucleic acid-binding proteins	Y	
38488	Nucleic acid-binding proteins	Y	
40961	Nucleic acid-binding proteins	Y	
34796	p53-like transcription factors	Y	Y
34855	p53-like transcription factors	Y	Y
35512	p53-like transcription factors	Y	Y
35525	p53-like transcription factors	Y	Y
36065	p53-like transcription factors	Y	Y
38119	p53-like transcription factors	Y	Y
38434	p53-like transcription factors	Y	Y
39062	p53-like transcription factors	Y	Y
41017	p53-like transcription factors	Y	
41370	p53-like transcription factors	Y	Y
44112	p53-like transcription factors	Y	Y
45029	p53-like transcription factors	Y	Y
45080	p53-like transcription factors	Y	Y
38425	SAND domain-like	Y	Y
41973	SAND domain-like	Y	Y
44332	SAND domain-like	Y	
40928	SMAD MH1 domain	Y	Y
41015	SRF-like	Y	
41235	SRF-like	Y	Y
43734	SRF-like	Y	Y
35524	STAT	Y	Y
35881	Transcriptional factor tubby, C-terminal domain	Y	
35957	Winged helix DNA-binding domain	Y	Y
35958	Winged helix DNA-binding domain	Y	Y
36285	Winged helix DNA-binding domain	Y	
36540	Winged helix DNA-binding domain	Y	Y
36707	Winged helix DNA-binding domain	Y	
37479	Winged helix DNA-binding domain	Y	
38516	Winged helix DNA-binding domain	Y	Y
38616	Winged helix DNA-binding domain	Y	Y
38992	Winged helix DNA-binding domain	Y	Y
39808	Winged helix DNA-binding domain	Y	
40891	Winged helix DNA-binding domain	Y	Y
45310	Winged helix DNA-binding domain	Y	Y
45339	Winged helix DNA-binding domain	Y	Y

Table S14. PFAM DNA binding domain list comparison with DBD Database (DBDdb)

Model ID	Model Name	Model Description	In DBD	In TFCat
PF05110	AF-4	AF-4 proto-oncoprotein	Y	Y
PF01586	Basic	Myogenic Basic domain	Y	
PF00170	bZIP_1	bZIP transcription factor	Y	Y
PF07716	bZIP_2	Basic region leucine zipper – bZIP_2	Y	Y
PF03131	bZIP_Maf	bZIP Maf transcription factor	Y	Y
PF02045	CBFB_NFYA	CCAAT-binding transcription factor (CBF-B/NF-YA) subunit B	Y	Y
PF00808	CBFD_NFYB_HMF	**Histone-like transcription factor (CBF/NF-Y) and archaeal histone**		Y
PF03859	CG-1	CG-1 domain	Y	
PF06573	Churchill	Churchill protein	Y	
PF04516	CP2	CP2 transcription factor	Y	Y
PF00313	CSD	'Cold-shock' DNA-binding domain	Y	
PF02376	CUT	CUT domain	Y	Y
PF02791	DDT	DDT domain	Y	Y
PF00751	DM	DM DNA binding domain	Y	
PF02319	E2F_TDP	E2F/DP family winged-helix DNA-binding domain	Y	Y
PF00178	Ets	Ets-domain	Y	Y
PF06818	Fez1	Fez1	Y	
PF00250	Fork_head	Fork head domain	Y	Y
PF00320	GATA	GATA zinc finger	Y	Y
PF03615	GCM	GCM motif protein	Y	Y
PF06320	GCN5L1	GCN5-like protein 1 (GCN5L1)	Y	
PF00010	HLH	Helix-loop-helix DNA-binding domain	Y	Y
PF00046	Homeobox	Homeobox domain	Y	Y
PF00447	HSF_DNA-bind	HSF-type DNA-binding	Y	Y
PF08279	HTH_11	HTH domain	Y	
PF01381	HTH_3	Helix-turn-helix	Y	
PF05225	HTH_psq	helix-turn-helix, Psq domain	Y	Y
PF00605	IRF	Interferon regulatory factor transcription factor	Y	Y
PF01056	Myc_N	Myc amino-terminal region	Y	
PF05224	NDT80_PhoG	NDT80 / PhoG like DNA-binding family	Y	
PF04054	Not1	CCR4-Not complex component, Not1	Y	
PF00870	P53	P53 DNA-binding domain	Y	Y
PF00292	PAX	'Paired box' domain	Y	Y
PF00157	Pou	Pou domain - N-terminal to homeobox domain	Y	Y
PF05044	Prox1	Homeobox prospero-like protein (PROX1)	Y	
PF02257	RFX_DNA_binding	RFX DNA-binding domain	Y	Y
PF00554	RHD	Rel homology domain (RHD)	Y	Y
PF00853	Runt	Runt domain	Y	Y
PF01342	SAND	SAND domain	Y	Y
PF03343	SART-1	SART-1 family	Y	
PF07093	SGT1	SGT1 protein	Y	
PF00319	SRF-TF	SRF-type transcription factor (DNA-binding and dimerisation domain)	Y	Y
PF02864	STAT_bind	STAT protein, DNA binding domain	Y	Y

Table S14. PFAM DNA binding domain list comparison with DBDdb (continued)

Model ID	Model Name	Model Description	In DBD	In TFCat
PF00907	T-box	T-box	Y	Y
PF01285	TEA	TEA/ATTS domain family	Y	Y
PF03299	TF_AP-2	Transcription factor AP-2	Y	Y
PF01167	Tub	Tub family	Y	
PF05764	YL1	YL1 nuclear protein	Y	
PF01754	zf-A20	A20-like zinc finger	Y	
PF02892	zf-BED	BED zinc finger	Y	
PF00096	zf-C2H2	Zinc finger, C2H2 type	Y	Y
PF01530	zf-C2HC	Zinc finger, C2HC type	Y	Y
PF00105	zf-C4	Zinc finger, C4 type (two domains)	Y	Y
PF02928	zf-C5HC2	C5HC2 zinc finger	Y	
PF06839	zf-GRF	GRF zinc finger	Y	
PF02891	zf-MIZ	MIZ zinc finger	Y	
PF01422	zf-NF-X1	NF-X1 type zinc finger	Y	
PF02135	zf-TAZ	TAZ zinc finger	Y	
PF01388	ARID	ARID/BRIGHT DNA binding domain		Y
PF02178	AT_hook	AT hook motif		Y
PF09270	Beta-trefoil	Beta-trefoil		Y
PF00859	CTF_NF1	CTF/NF-I family transcription modulation region		Y
PF02946	GTF2I	GTF2I-like repeat		Y
PF00505	HMG_box	HMG (high mobility group) box		Y
PF09271	LAG1-DNAbind	LAG1, DNA binding		Y
PF01429	MBD	Methyl-CpG binding domain		Y
PF03165	MH1	MH1 domain		Y
PF00249	Myb_DNA-binding	Myb-like DNA-binding domain		Y
PF09111	SLIDE	SLIDE		Y
PF00352	TBP	Transcription factor TFIID (or TATA-binding protein, TBP)		Y
PF03529	TF_Otx	Otx1 transcription factor		Y
PF02008	zf-CXXC	CXXC zinc finger domain		Y

Table S15. Fox family gene test set

Gene ID	Gene Symbol	Gene Description
15375	Foxa1	forkhead box A1
15376	Foxa2	forkhead box A2
15377	Foxa3	forkhead box A3
64290	Foxb1	forkhead box B1
14240	Foxb2	forkhead box B2
17300	Foxc1	forkhead box C1
14234	Foxc2	forkhead box C2
15229	Foxd1	forkhead box D1
17301	Foxd2	forkhead box D2
15221	Foxd3	forkhead box D3
14237	Foxd4	forkhead box D4
110805	Foxe1	forkhead box E1
30923	Foxe3	forkhead box E3
15227	Foxf1a	forkhead box F1a
14238	Foxf2	forkhead box F2
15228	Foxg1	forkhead box G1
14106	Foxh1	forkhead box H1
14233	Foxi1	forkhead box I1
270004	Foxi2	forkhead box I2
15223	Foxj1	forkhead box J1
60611	Foxj2	forkhead box J2
230700	Foxj3	forkhead box J3
17425	Foxk1	forkhead box K1
68837	Foxk2	forkhead box K2
14241	Foxl1	forkhead box L1
26927	Foxl2	forkhead box L2
14235	Foxm1	forkhead box M1
15218	Foxn1	forkhead box N1
14236	Foxn2	forkhead box N2
71375	Foxn3	forkhead box N3
116810	Foxn4	forkhead box N4
56458	Foxo1	forkhead box O1
56484	Foxo3a	forkhead box O3a
54601	Foxo4	forkhead box O4
329934	Foxo6	forkhead box O6
108655	Foxp1	forkhead box P1
114142	Foxp2	forkhead box P2
20371	Foxp3	forkhead box P3
74123	Foxp4	forkhead box P4
15220	Foxq1	forkhead box Q1

Table S16. Sox family gene test set

Gene ID	Gene Symbol	Gene Description
20665	Sox10	SRY-box containing gene 10
20666	Sox11	SRY-box containing gene 11
20664	Sox1	SRY-box containing gene 1
20667	Sox12	SRY-box containing gene 12
20668	Sox13	SRY-box containing gene 13
20669	Sox14	SRY-box containing gene 14
20670	Sox15	SRY-box containing gene 15
20671	Sox17	SRY-box containing gene 17
20672	Sox18	SRY-box containing gene 18
223227	Sox21	SRY-box containing gene 21
20674	Sox2	SRY-box containing gene 2
214105	Sox30	SRY-box containing gene 30
20675	Sox3	SRY-box containing gene 3
20677	Sox4	SRY-box containing gene 4
20678	Sox5	SRY-box containing gene 5
20679	Sox6	SRY-box containing gene 6
20680	Sox7	SRY-box containing gene 7
20681	Sox8	SRY-box containing gene 8
20682	Sox9	SRY-box containing gene 9
21674	Sry	sex determining region of Chr Y

Additional Data File 1: Figures

Figure S1. Overlap of initial datasets. The gene identifiers were evaluated for overlap in both the Union of Putative TFs (UPTF) (Venn diagram Figure S1 Panel A) and Transcription Factor Candidates (TFC) (Venn diagram Figure S1 Panel B) sets.

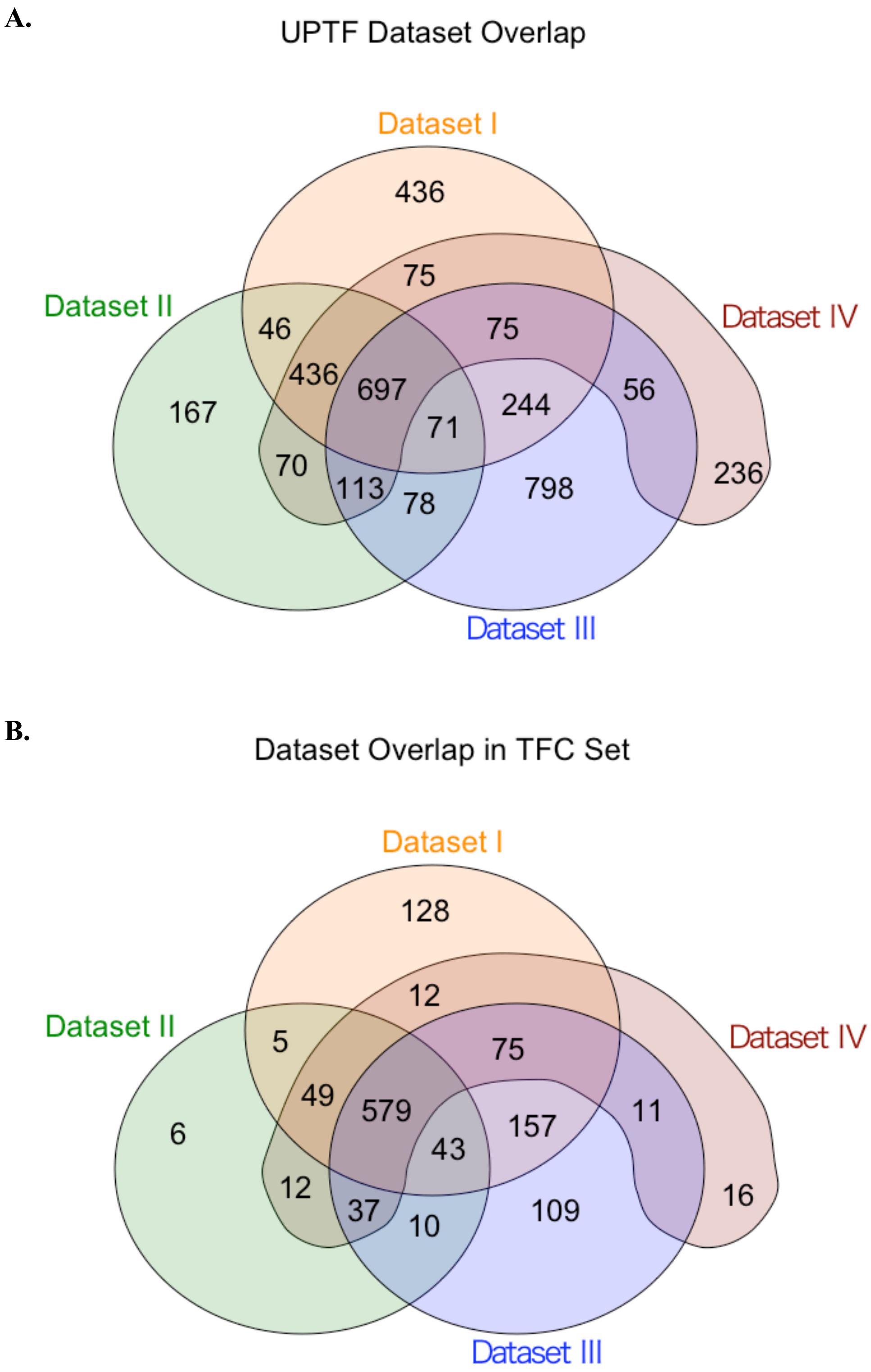

Figure S2. Analysis of cluster pruning methods using the Fox test set: plots of cluster sensitivity (proportion of members of the Fox test set in a cluster), cluster specificity (number of cluster members that are members of the Fox test set), and cluster size across increasing I'_s derived from two different pruning methods. Each line in a panel represents the evaluation of one cluster containing one or more Fox test set genes. Analysis for each specific cluster is represented by the same line color and point symbol combination across all plots in the test set evaluation (some line attributes cannot be easily distinguished when they overlap on a plot). Panel A: Clusters sensitivity values for Fox clusters across increasing I'_s when clusters are pruned using only I'_s thresholds (x-axis). Panel B: Cluster specificity values for Fox clusters across increasing I'_s when clusters are pruned using only I'_s value thresholds. Panel C: Resulting cluster sizes (cardinalities) for Fox clusters across increasing I'_s when clusters are pruned using only I'_s value thresholds. Panel D: Cluster sensitivity values for Fox clusters across increasing I'_s (x-axis) when clusters are pruned using domain-matching as a primary criteria and I'_s thresholds applied secondarily when domain matching criteria is not satisfied. Panel E: Cluster specificity values for Fox clusters across increasing I'_s when clusters are pruned using domain-matching as a primary criteria and I'_s thresholds applied secondarily when domain matching criteria is not satisfied. Panel F: Resulting cluster sizes for Fox clusters across increasing I'_s when clusters are pruned using domain-matching as a primary criteria and I'_s thresholds applied secondarily when domain matching criteria is not satisfied.

A.

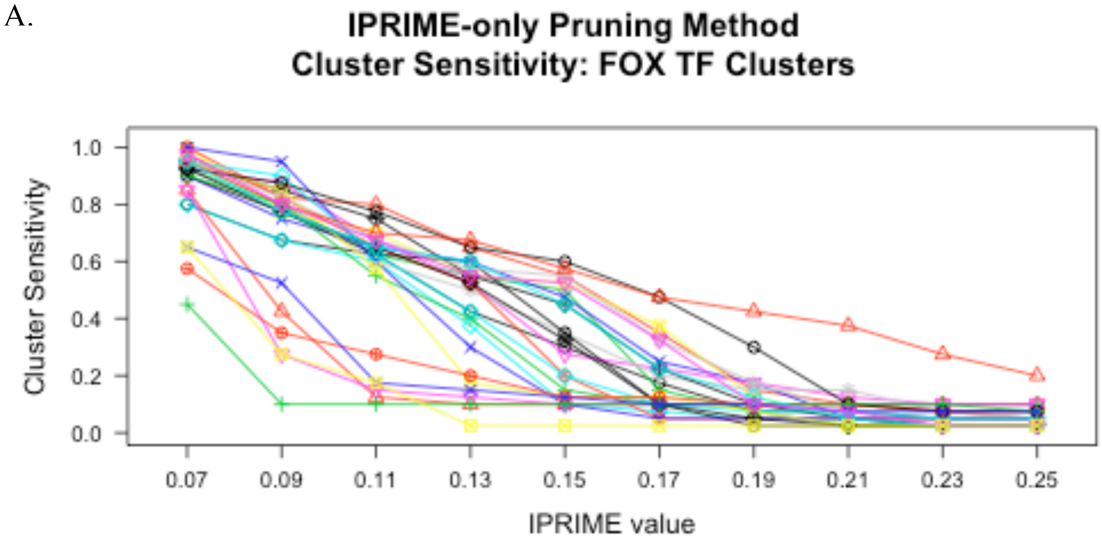

IPRIME-only Pruning Method
Cluster Sensitivity: FOX TF Clusters
Cluster Sensitivity
1.0
0.8
0.6
0.4
0.2
0.0
0.07
0.09
0.11
0.13
0.15
0.17
0.19
0.21
0.23
0.25
IPRIME value

B.

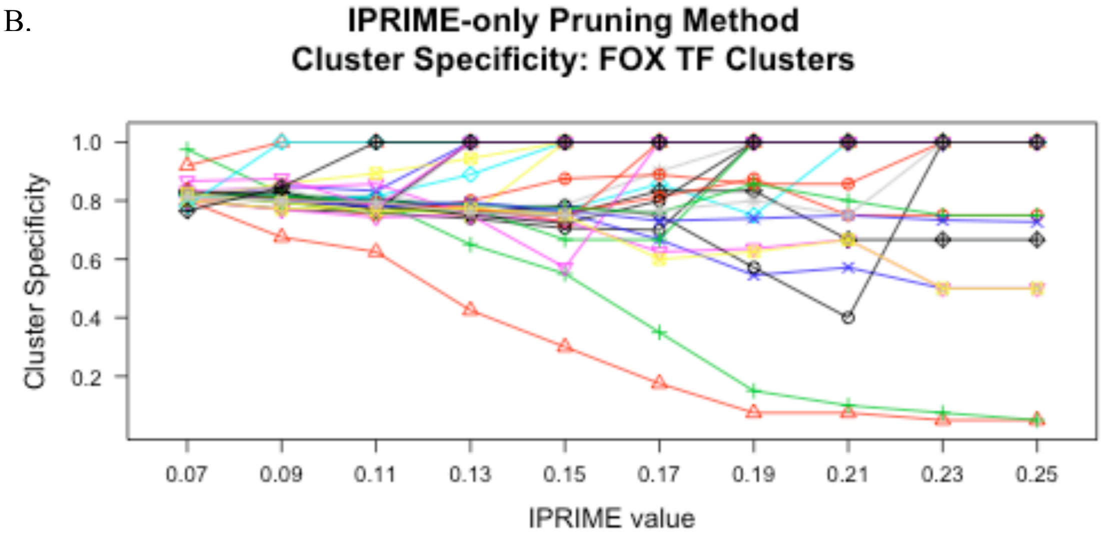

IPRIME-only Pruning Method
Cluster Specificity: FOX TF Clusters
Cluster Specificity
1.0
0.8
0.6
0.4
0.2
0.07
0.09
0.11
0.13
0.15
0.17
0.19
0.21
0.23
0.25
IPRIME value

C.

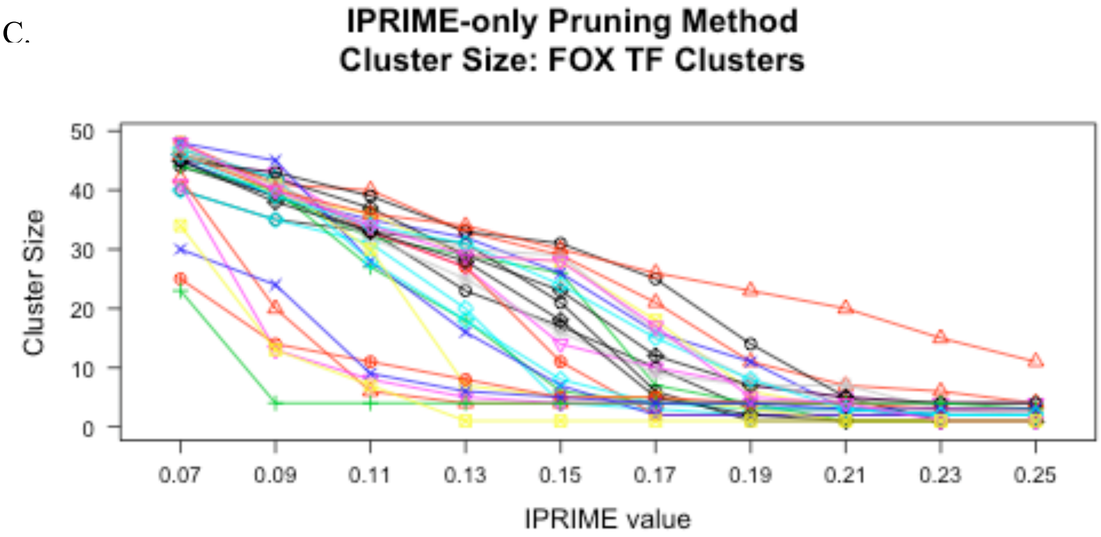

IPRIME-only Pruning Method
Cluster Size: FOX TF Clusters
Cluster Size
50
40
30
20
10
0
0.07
0.09
0.11
0.13
0.15
0.17
0.19
0.21
0.23
0.25
IPRIME value

Domain-based Pruning Method
Cluster Sensitivity: FOX TF Clusters

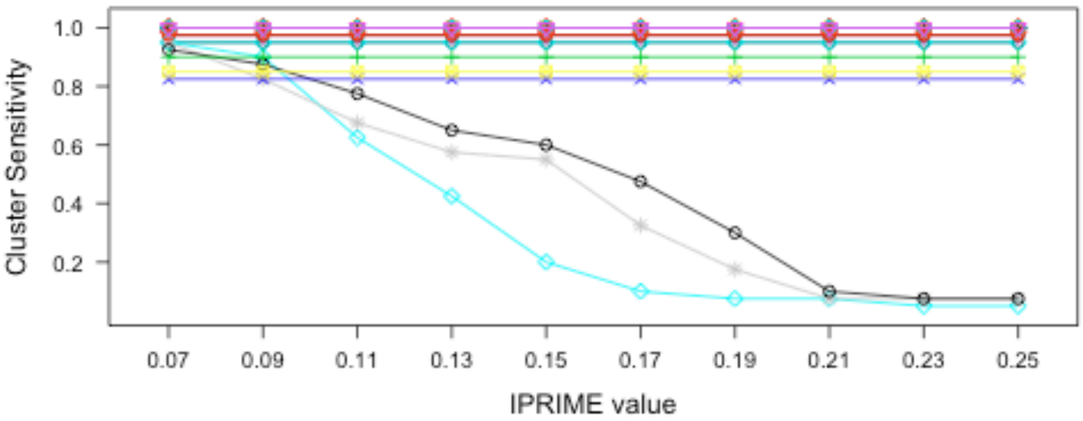

Domain-based Pruning Method
Cluster Specificity: FOX TF Clusters

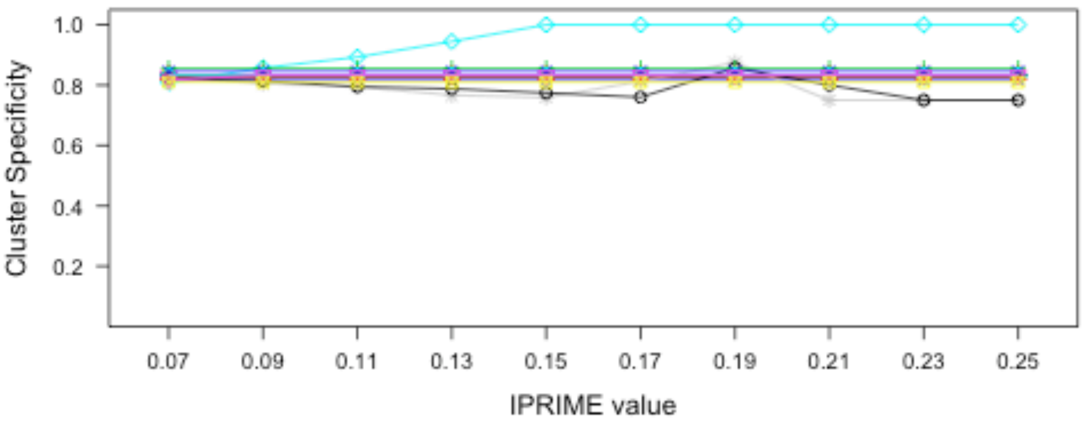

Domain-based Pruning Method
Cluster Size: FOX TF Clusters

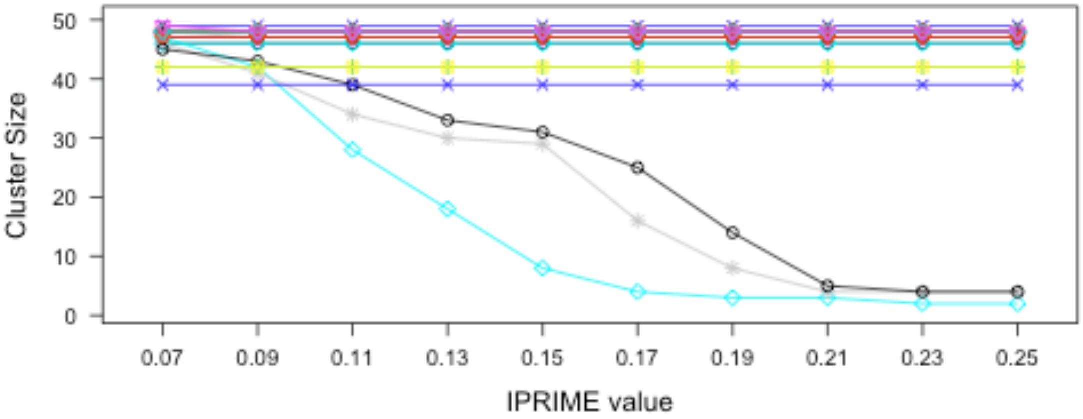

Figure S3. Analysis of cluster pruning methods using the Sox test set: plots of cluster sensitivity (proportion of members of the Sox test set in a cluster), cluster specificity (number of cluster members that are members of the Sox test set), and cluster size across increasing I'_s derived from two different pruning methods. Each line in a panel represents the evaluation of one cluster containing one or more Sox test set genes. Analysis for each specific cluster is represented by the same line color and point symbol combination across all plots in the test set evaluation (some line attributes cannot be easily distinguished when they overlap on a plot). Panel A: Clusters sensitivity values for Sox clusters across increasing I'_s when clusters are pruned using only I'_s thresholds (x-axis). Panel B: Cluster specificity values for Sox clusters across increasing I'_s when clusters are pruned using only I'_s value thresholds. Panel C: Resulting cluster sizes (cardinalities) for Sox clusters across increasing I'_s when clusters are pruned using only I'_s value thresholds. Panel D: Cluster sensitivity values for Sox clusters across increasing I'_s when clusters are pruned using domain-matching as a primary criteria and I'_s thresholds applied secondarily when domain matching criteria is not satisfied. Panel E: Cluster specificity values for Sox clusters across increasing I'_s when clusters are pruned using domain-matching as a primary criteria and I'_s thresholds applied secondarily when domain matching criteria is not satisfied. Panel F: Resulting cluster sizes for Sox clusters across increasing I'_s when clusters are pruned using domain-matching as a primary criteria and I'_s thresholds applied secondarily when domain matching criteria is not satisfied.

A.

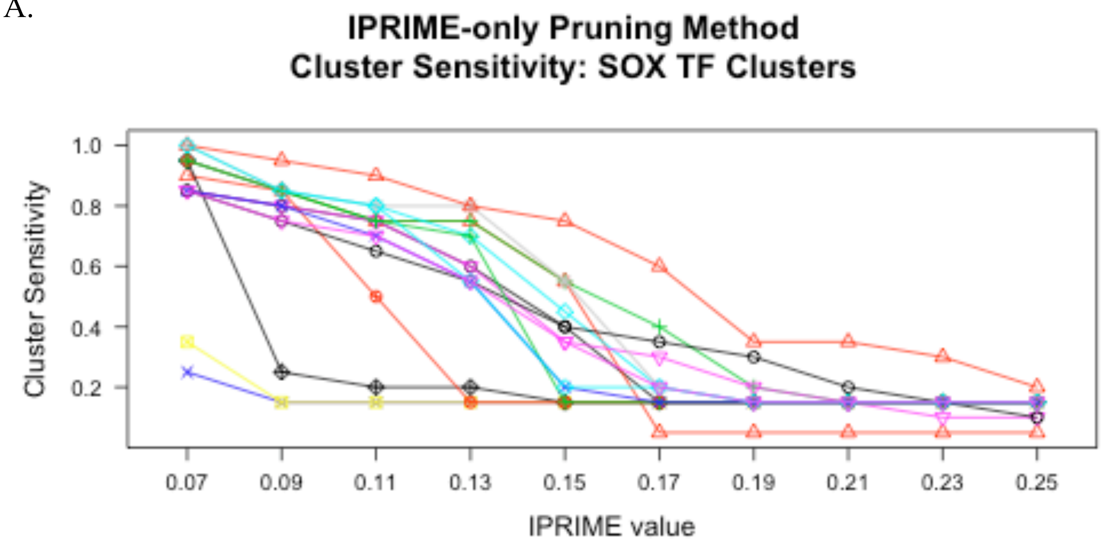
IPRIME-only Pruning Method
Cluster Sensitivity: SOX TF Clusters
Cluster Sensitivity
1.0
0.8
0.6
0.4
0.2
0.07 0.09 0.11 0.13 0.15 0.17 0.19 0.21 0.23 0.25
IPRIME value

B.

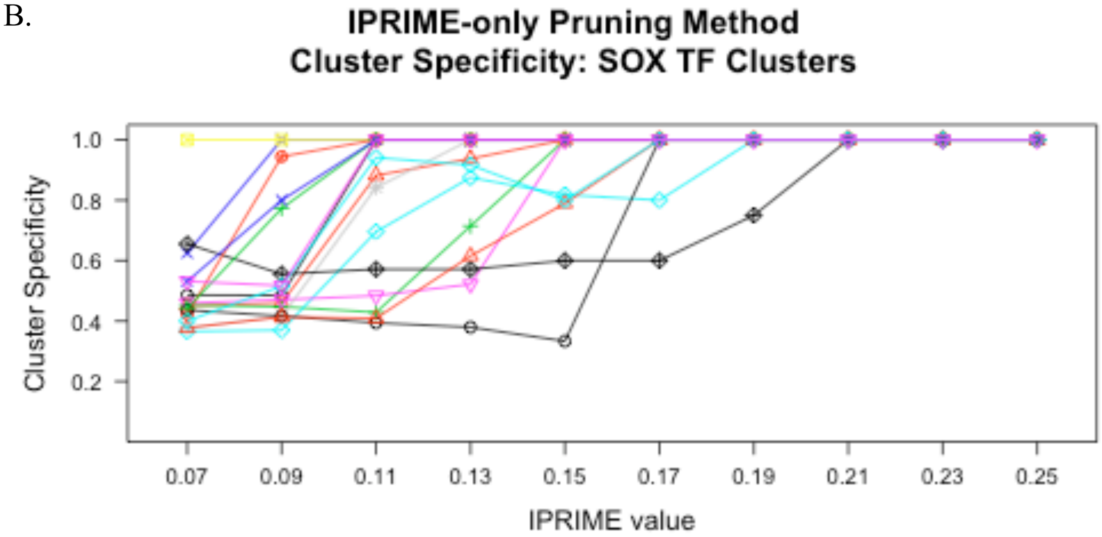
IPRIME-only Pruning Method
Cluster Specificity: SOX TF Clusters
Cluster Specificity
1.0
0.8
0.6
0.4
0.2
0.07 0.09 0.11 0.13 0.15 0.17 0.19 0.21 0.23 0.25
IPRIME value

C.

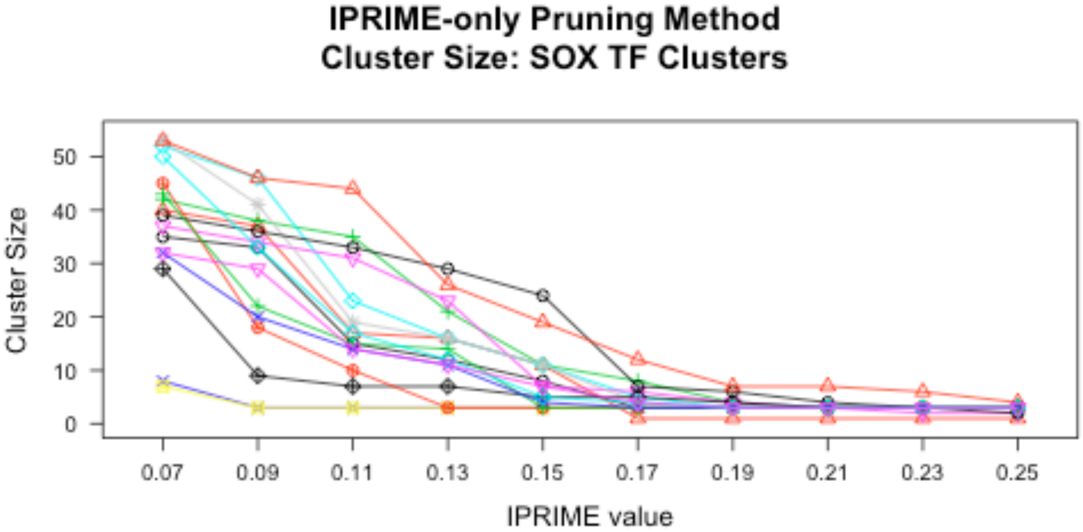
IPRIME-only Pruning Method
Cluster Size: SOX TF Clusters
Cluster Size
50
40
30
20
10
0
0.07 0.09 0.11 0.13 0.15 0.17 0.19 0.21 0.23 0.25
IPRIME value

D.

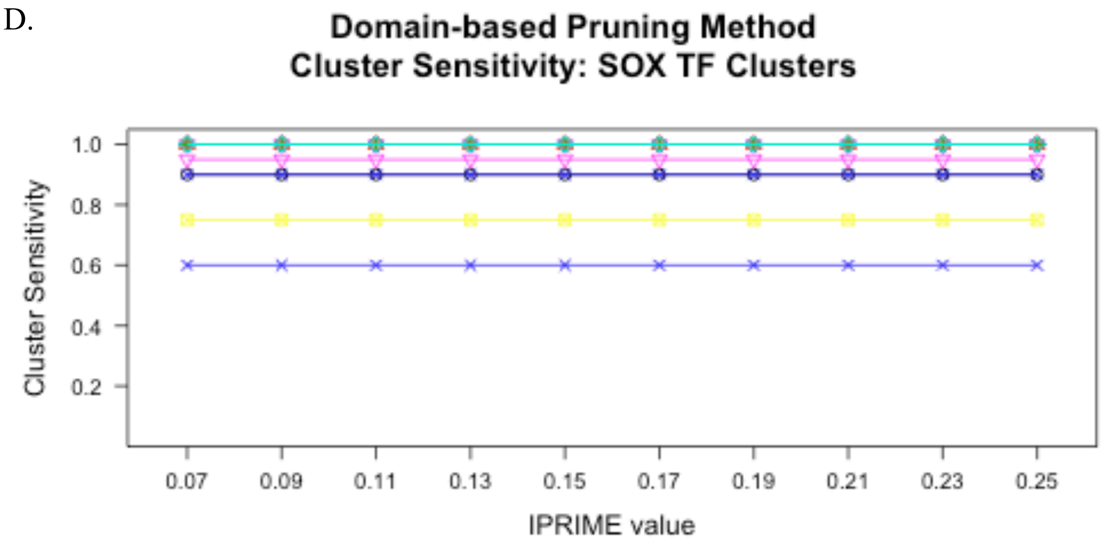
Domain-based Pruning Method
Cluster Sensitivity: SOX TF Clusters
Cluster Sensitivity
1.0
0.8
0.6
0.4
0.2
0.07
0.09
0.11
0.13
0.15
0.17
0.19
0.21
0.23
0.25
IPRIME value

E.

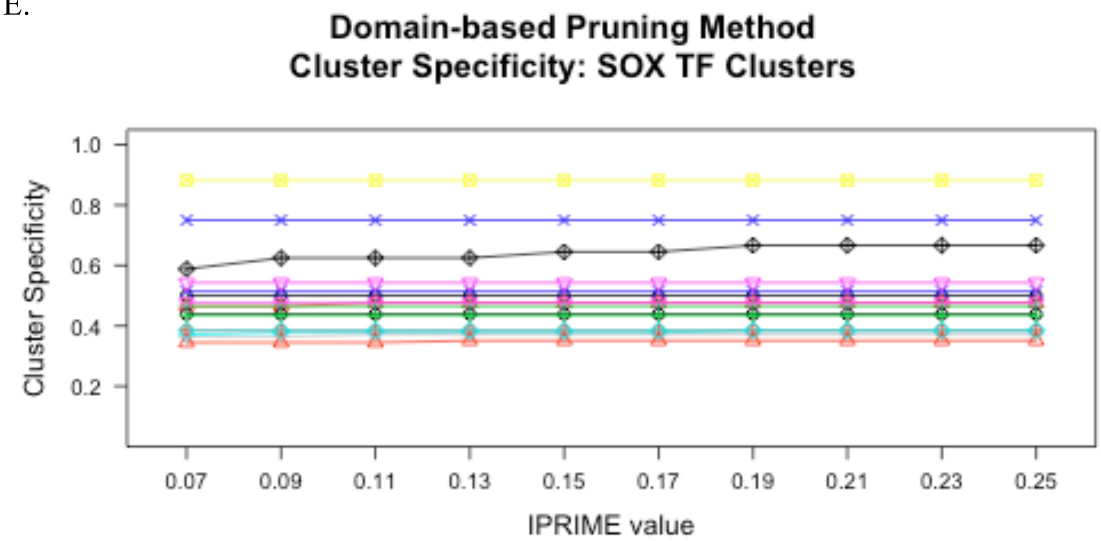
Domain-based Pruning Method
Cluster Specificity: SOX TF Clusters
Cluster Specificity
1.0
0.8
0.6
0.4
0.2
0.07
0.09
0.11
0.13
0.15
0.17
0.19
0.21
0.23
0.25
IPRIME value

F.

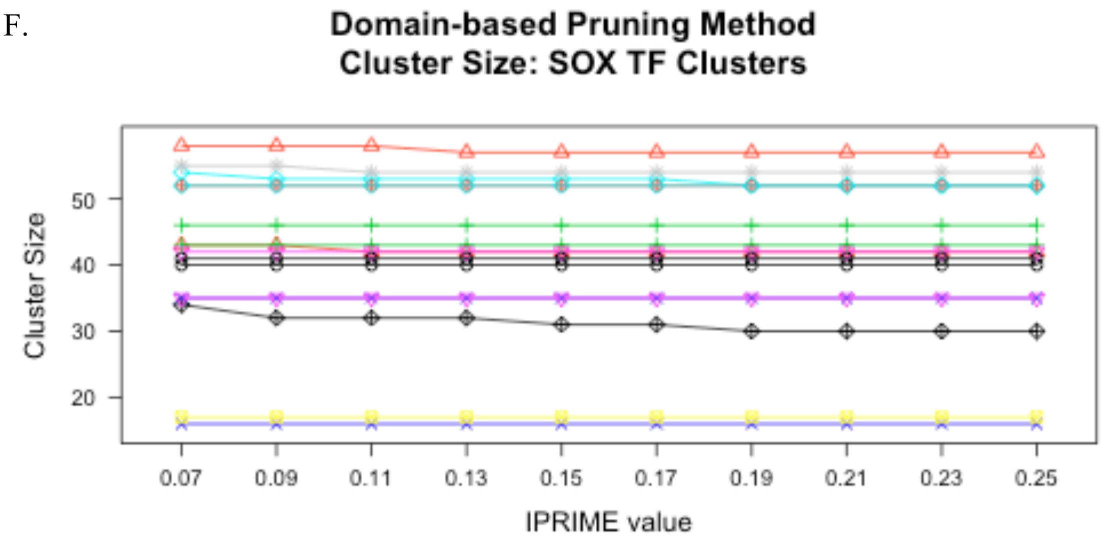
Domain-based Pruning Method
Cluster Size: SOX TF Clusters
Cluster Size
50
40
30
20
0.07
0.09
0.11
0.13
0.15
0.17
0.19
0.21
0.23
0.25
IPRIME value

Figure S4. TFCat annotation workflow

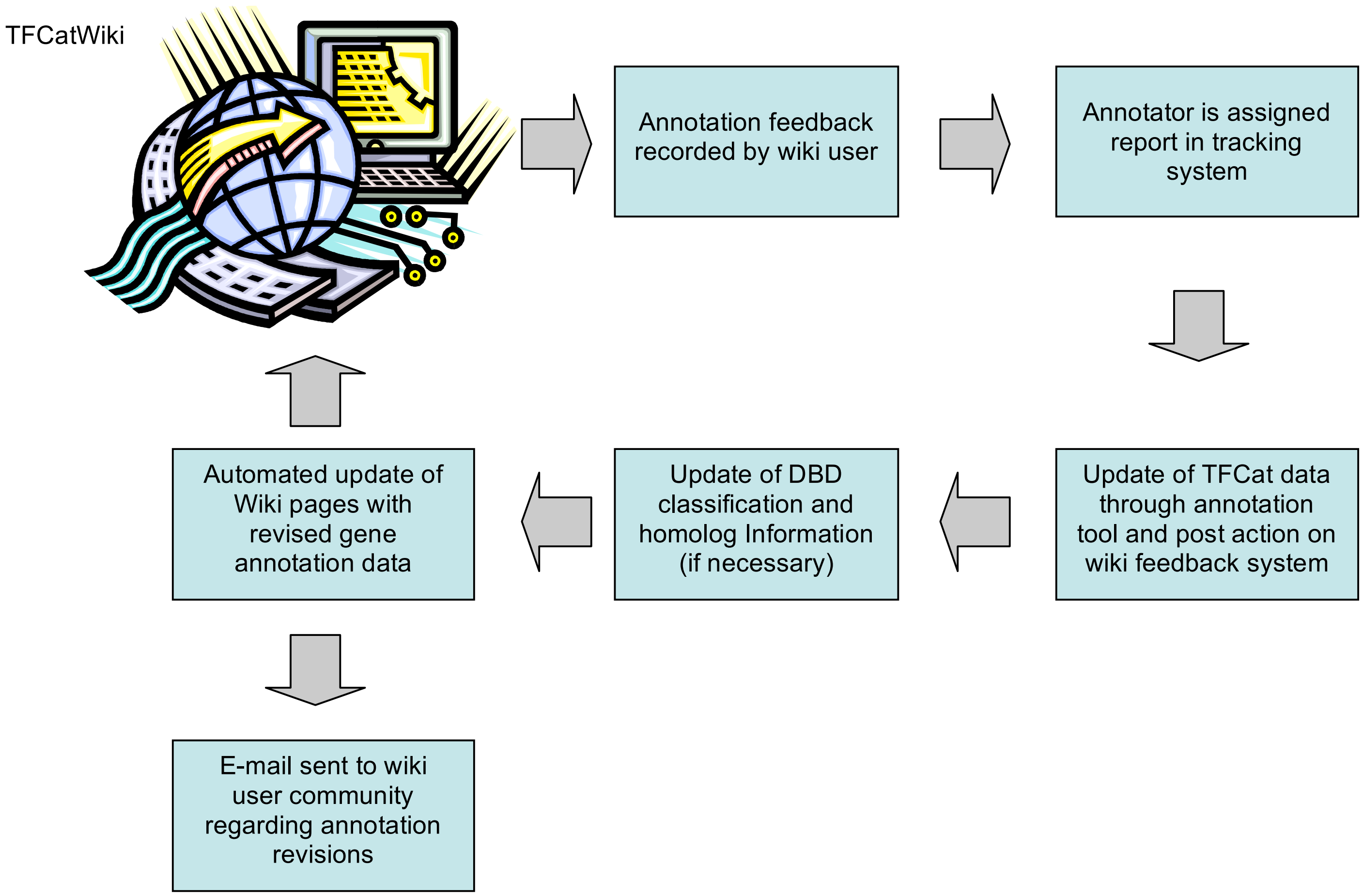

Figure S5. Screen shots of the backend web-based TFCat Annotation Tool. Panel A: Each reviewer has password-protected access to a full or partial list of genes assigned to their annotation queue. Panel B: One or more genes may be selected for annotation review/update. Panel C: The gene annotation page facilitates recording of PubMed article reviews and entry of functional taxa and TF judgment assignments. The tool also provides direct access to additional web-based gene information and literature resources to facilitate the curation.

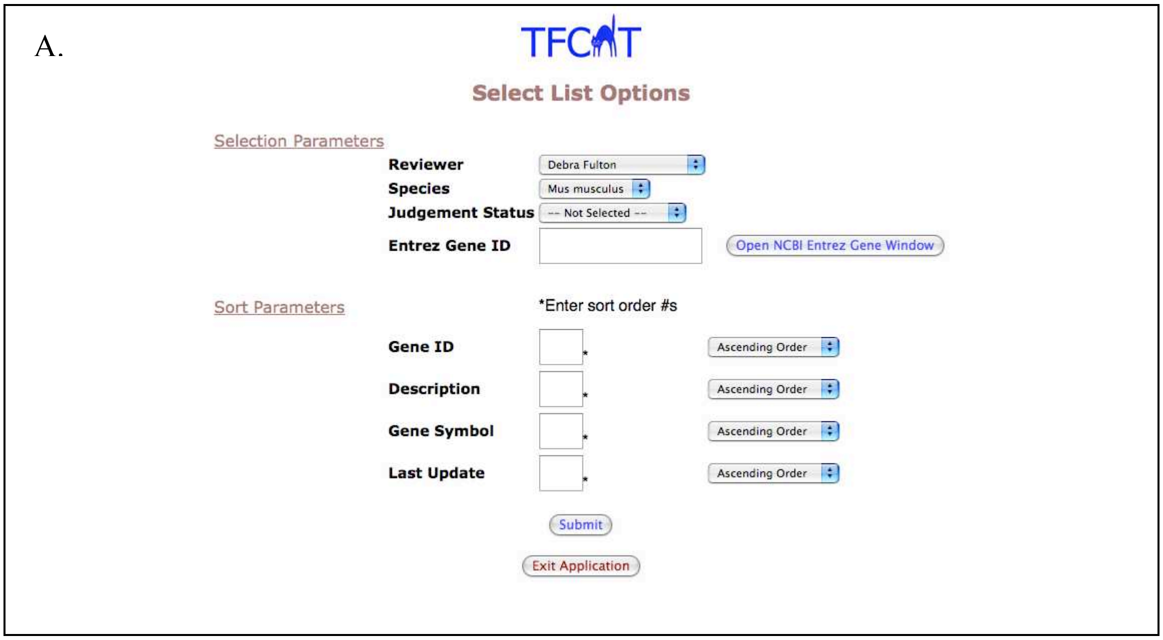

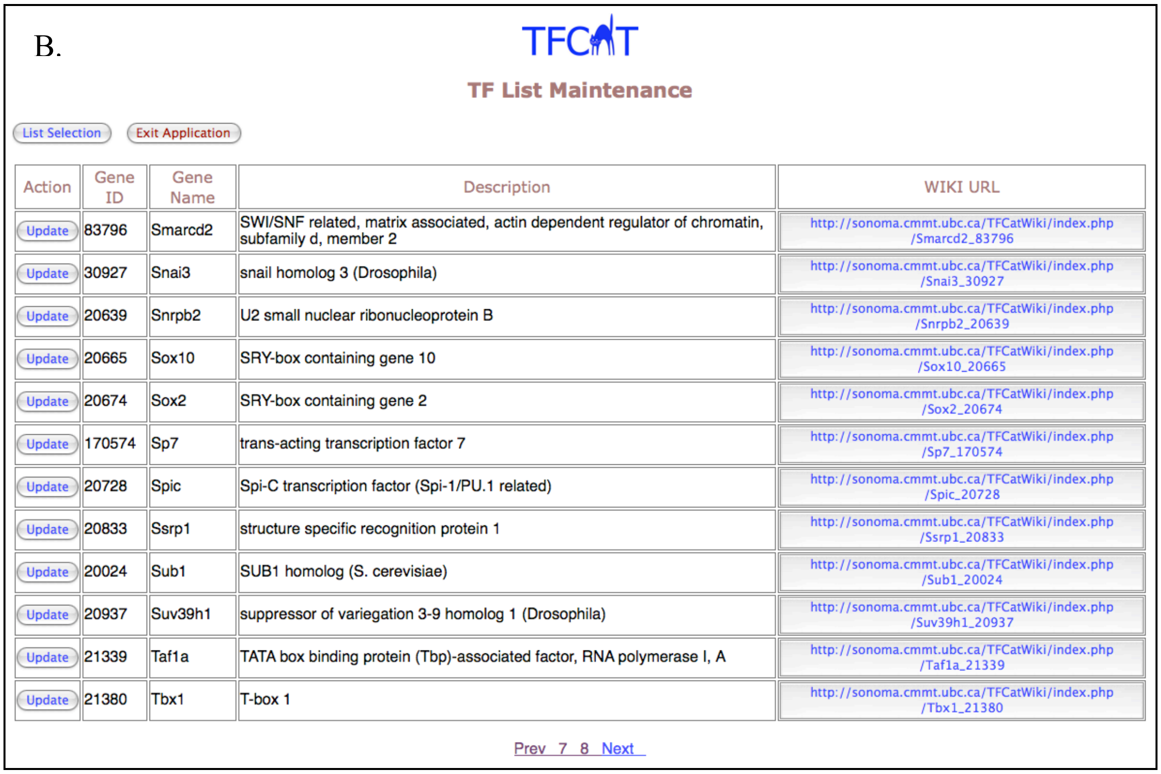

Action	Gene ID	Gene Name	Description	WIKI URL
Update	83796	Smarcd2	SWI/SNF related, matrix associated, actin dependent regulator of chromatin, subfamily d, member 2	http://sonoma.cmmt.ubc.ca/TFCatWiki/index.php/Smarcd2_83796
Update	30927	Snai3	snail homolog 3 (Drosophila)	http://sonoma.cmmt.ubc.ca/TFCatWiki/index.php/Snai3_30927
Update	20639	Snrpb2	U2 small nuclear ribonucleoprotein B	http://sonoma.cmmt.ubc.ca/TFCatWiki/index.php/Snrpb2_20639
Update	20665	Sox10	SRY-box containing gene 10	http://sonoma.cmmt.ubc.ca/TFCatWiki/index.php/Sox10_20665
Update	20674	Sox2	SRY-box containing gene 2	http://sonoma.cmmt.ubc.ca/TFCatWiki/index.php/Sox2_20674
Update	170574	Sp7	trans-acting transcription factor 7	http://sonoma.cmmt.ubc.ca/TFCatWiki/index.php/Sp7_170574
Update	20728	Spic	Spi-C transcription factor (Spi-1/PU.1 related)	http://sonoma.cmmt.ubc.ca/TFCatWiki/index.php/Spic_20728
Update	20833	Ssrp1	structure specific recognition protein 1	http://sonoma.cmmt.ubc.ca/TFCatWiki/index.php/Ssrp1_20833
Update	20024	Sub1	SUB1 homolog (S. cerevisiae)	http://sonoma.cmmt.ubc.ca/TFCatWiki/index.php/Sub1_20024
Update	20937	Suv39h1	suppressor of variegation 3-9 homolog 1 (Drosophila)	http://sonoma.cmmt.ubc.ca/TFCatWiki/index.php/Suv39h1_20937
Update	21339	Taf1a	TATA box binding protein (Tbp)-associated factor, RNA polymerase I, A	http://sonoma.cmmt.ubc.ca/TFCatWiki/index.php/Taf1a_21339
Update	21380	Tbx1	T-box 1	http://sonoma.cmmt.ubc.ca/TFCatWiki/index.php/Tbx1_21380

C.

TFCAT

Maintain Gene Assessment Information

Update Gene Entry | Go To Selected TF List
Open Reviewer Guidelines/Help Window

TF Gene Candidate Information

Gene ID: 20674 — Open NCBI Entrez Gene Window | Open IHOP Window | Open SWISSPROT Window

Description: Sox2 SRY-box containing gene 2

Gene Aliases: lcc; Sox-2; ysb;

GeneRIF Information

PubMed IDs		GeneRIF
View	12167158	role in regulating lens-specific delta1-crystallin enhancer
View	12923055	Sox2 can dimerize onto DNA in a distinct conformational arrangement.
View	15113840	newly identified enhancer with Sox elements activates the alphaB-crystallin promoter in the lens, although they are separated by the entire HSPB2 gene
View	15121842	Sox-2 regulatory region 2 is the first example of an enhancer in which a single regulatory core sequence is involved in multipotent-state-specific expression in two different stem cells
View	15240551	These findings highlight a crucial and unexpected role for Sox2 in the maintenance of neurones in selected brain areas.
View	15262984	findings indicate a role for the same POU binding motifs in Sox2 transgene regulation in both ES and neural precursor cells
View	15557334	results indicate that Oct-3/4 is a member of the gene family regulated by Oct-3/4 and Sox2
View	15695336	One of the abnormally elevated genes in Egr2Lo/Lo Schwann cells, Sox2, encodes a transcription factor that is crucial for maintenance of neural stem cell pluripotency.
View	15711057	These studies demonstrate that SOX2 may meet the requirements of a universal neural stem cell marker and provides a means to identify cells which fulfill the basic criteria of a stem cell: self-renewal and multipotent differentiation.
View	15846349	absence or reduced expression of the transcription factor SOX2 within the developing inner ear, results in inner ear malformation
View	15860457	nanog is transcriptionally regulated by OCT4 and SOX2
View	15863505	Sox15 and Sox2 have distinct roles in transcriptional control in mouse ES cells
View	16026334	Expression of Sox2 is a unifying characteristic of neural stem cells in the adult rat brain, but that not all NSCs maintain the ability to form all neural cell types in vivo.

PubMed Evidence

	PubMed IDs	Function	Species	Evidence Strength	Comments
Maintain	15860457	DNA Binding; Transactivation;	Human; Mouse;	Strong	Mutations affecting the Oct and Sox elements in the Nanog promoter reduced the activity of a luciferase reporter gene. Oct4 and Sox2 bind to the Nanog promoter by ChIP experiments and supershift EMSA assays. Knockdown of Oct4 or Sox2 with RNAi reduces Nanog promoter-luciferase reporter gene activity.

Title: Transcriptional regulation of nanog by OCT4 and SOX2.

Add PubMed Information

Reviewer's Assessment

Reviewer's Judgement Previously Selected Judgement: TF Gene

TF Gene

Taxonomy Previously Selected Taxa: DNA-Binding: sequence-specific;

Transcription Regulatory Activity: heterochromatin interaction/binding
DNA-Binding: sequence-specific
DNA-Binding: non-sequence-specific
Single stranded RNA/DNA binding
Transcription Factor Binding: tf co-factor binding
Basal Transcription Factor

Reviewer Comments

Sox2 is a transcription factor. It binds DNA by ChIP and supershift EMSA assays and is required for Nanog promoter activity by luciferase reporter gene and RNAi assays.

TFCatWiki URL http://sonoma.cmmt.ubc.ca/TFCatWiki/index.php/Sox2_20674

Open TFCatWiki Window

Update Gene Entry | Go To Selected TF List | Exit Application
Open Reviewer Guidelines/Help Window

Figure S6. Final cluster membership for the Sox containing test set genes. Genes that are members of the test set are colored in yellow. Panel A: Final Sox cluster membership when domain-matching as a primary criteria and I'_s thresholds applied secondary criteria is applied, in conjunction with an approximation method for merging proportionally linked clusters. Panel B: Example of final Sox containing merged clusters when only the I'_s threshold method is applied (using an I'_s value of 0.21)

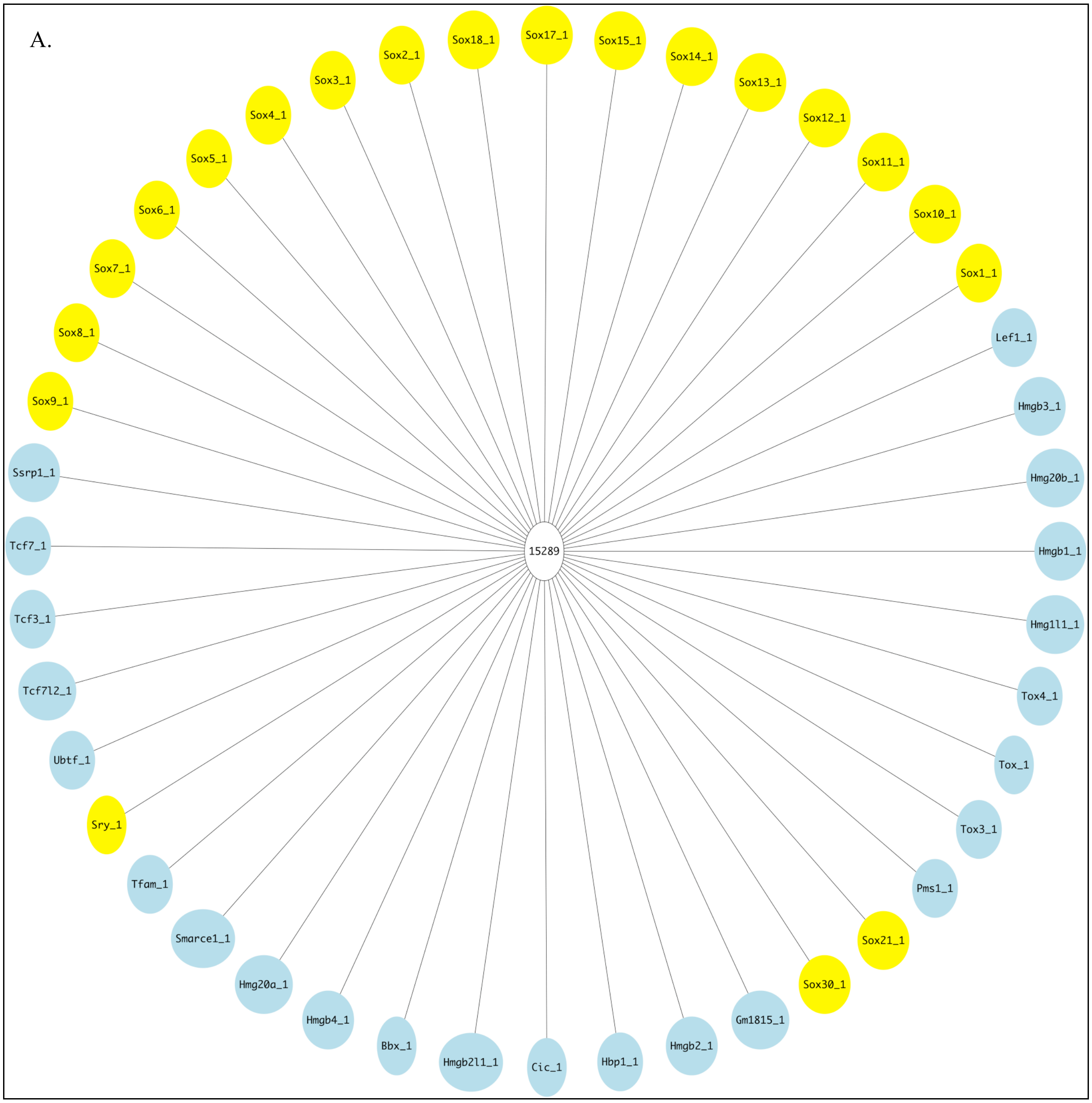

B.

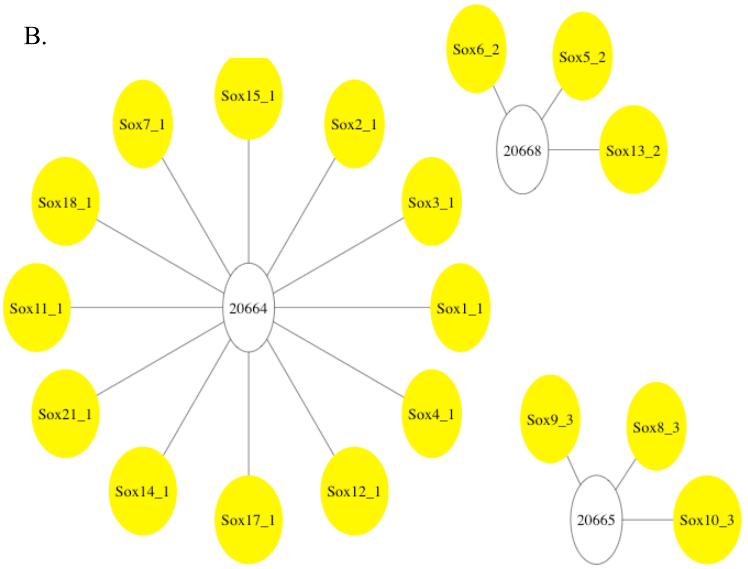

Sox15_1
Sox7_1
Sox2_1
Sox18_1
Sox3_1
Sox11_1
20664
Sox1_1
Sox21_1
Sox4_1
Sox14_1
Sox17_1
Sox12_1
Sox6_2
Sox5_2
20668
Sox13_2
Sox9_3
Sox8_3
20665
Sox10_3

Figure S7. Final cluster membership for the Fox containing test set genes. Genes that are members of the test set are colored in yellow. Panel A: Final Fox cluster membership when domain-matching as a primary criteria and I'_s thresholds applied secondary criteria is applied, in conjunction with an approximation method for merging proportionally linked clusters. Panel B: Example of final Fox merged clusters when only the I'_s threshold method is applied (using an I'_s value of 0.21).

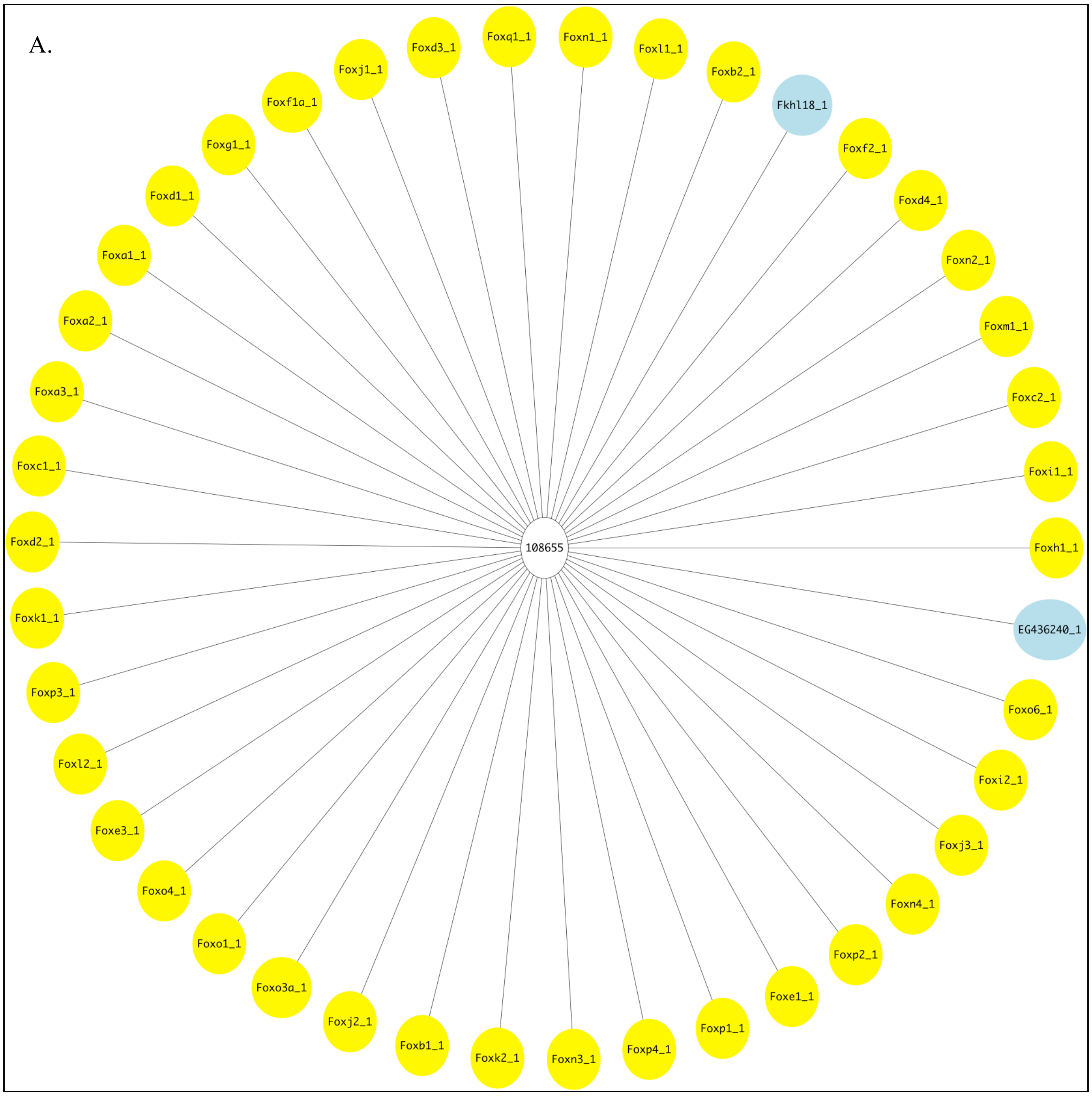

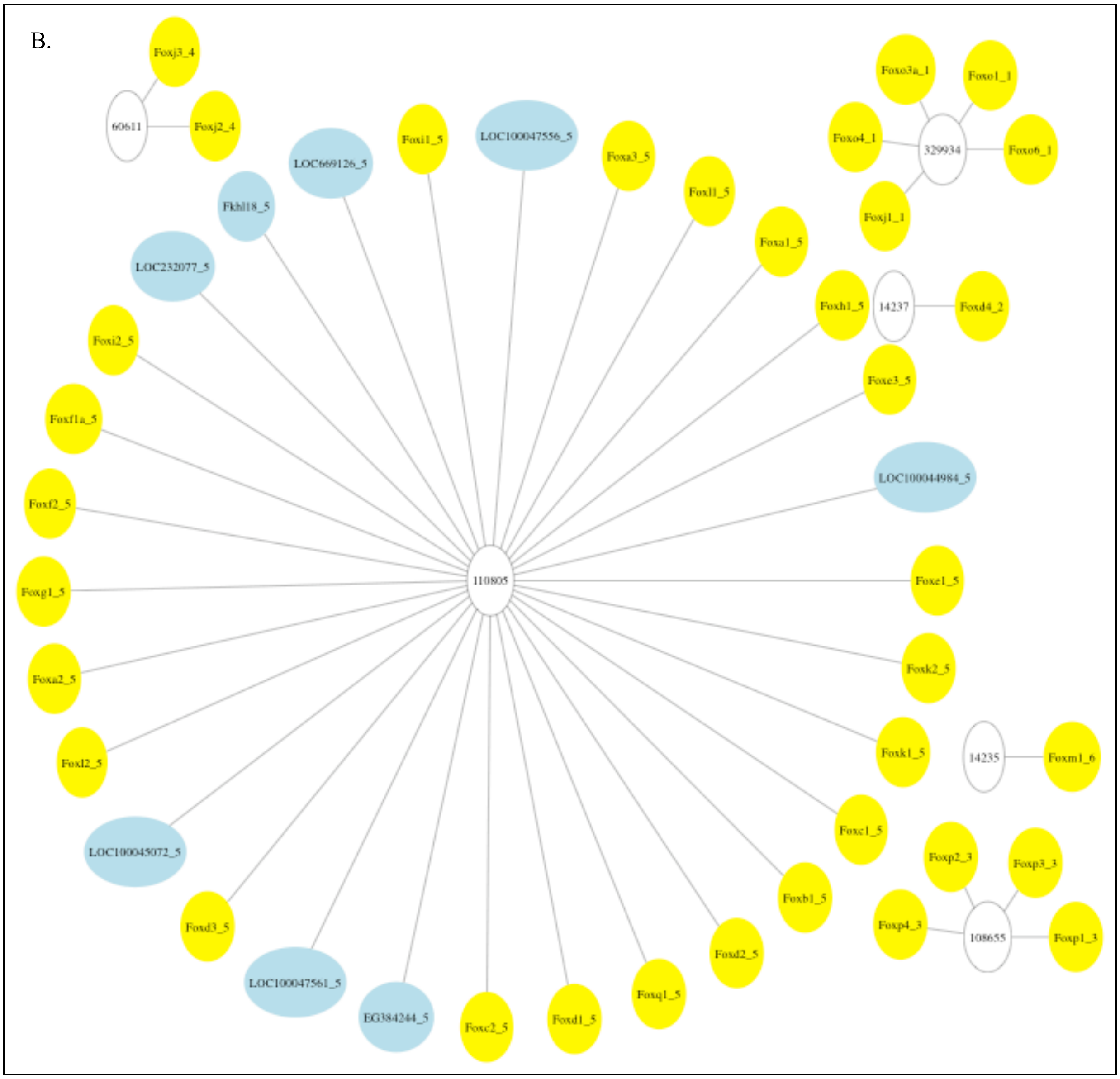
B.
Foxj3_4
60611
Foxj2_4
Foxo3a_1
Foxo1_1
Foxo4_1
329934
Foxo6_1
Foxj1_1
LOC669126_5
Foxi1_5
LOC100047556_5
Foxa3_5
Foxl1_5
Fkhl18_5
LOC232077_5
Foxa1_5
Foxh1_5
14237
Foxd4_2
Foxi2_5
Foxe3_5
Foxf1a_5
LOC100044984_5
Foxf2_5
110805
Foxg1_5
Foxe1_5
Foxa2_5
Foxk2_5
Foxl2_5
Foxk1_5
14235
Foxm1_6
LOC100045072_5
Foxc1_5
Foxp2_3
Foxp3_3
Foxb1_5
Foxd3_5
Foxp4_3
108655
Foxp1_3
Foxd2_5
LOC100047561_5
Foxq1_5
EG384244_5
Foxc2_5
Foxd1_5

Figure S8. Example of pruned Fox-containing clusters generated using the I'_s only method using a threshold of 0.21.

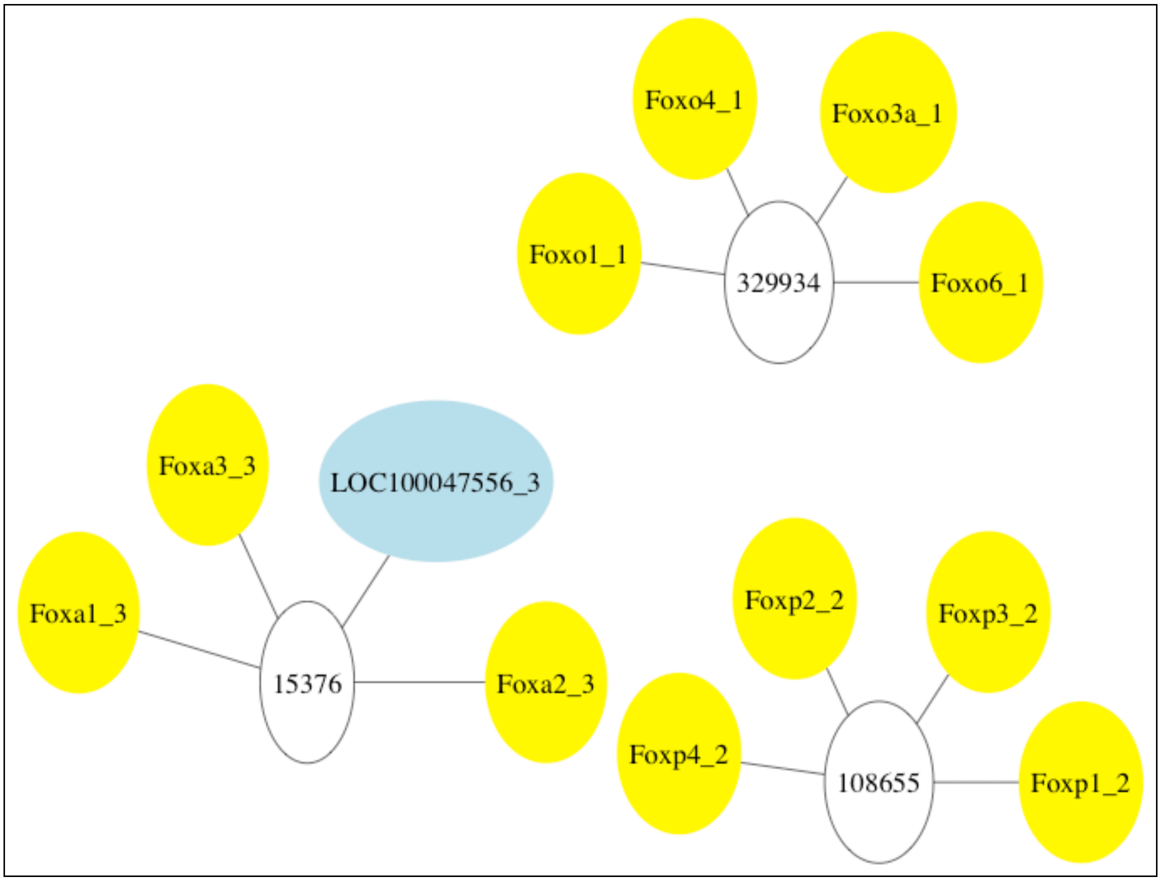

Supplement: Additional data file 1 — Table S1: gene annotation judgment summary counts from the quality assurance assessment process. Table S2: quality assurance gene pair judgment annotations. Table S3: independent annotations of TFs when the same PubMed evidence was used. Table S4: PFAM and Superfamily group model DBD predictions for the annotated TF genes. Table S5: DNA-binding classification extensions added to the Luscombe et al. [15] classification system. Table S6: protein class counts of genes predicted to contain multiple instances of the same DBD. Table S7: DNA-binding TFs predicted to contain two different DNA-binding classes. Table S8: DNA-binding TFs that do not contain a detected DBD. Table S9: DNA-binding TFs with no detected protein family-level domain. Table S10: annotated single-stranded DNA-binding TFs. Table S11: summary of the counts enumerated in the TFCat comparison with GO. Table S12: summary of the counts enumerated in the comparison of TFCat classified HMM DBDs with the DBD database (DBDdb). Table S13: Superfamily DBD comparisons with DBDdb. Table S14: PFAM DBD comparisons with DBDdb. Table S15: Fox family test set genes. Table S16: Sox family test set genes. Figure S1: Venn diagram of the overlap of the four initial TF datasets. Figure S2: plots for the analysis of cluster pruning methods using the Fox test set. Figure S3: plots for the analysis of cluster pruning methods using the Sox test set. Figure S4: TFCat annotation workflow implementation. Figure S5: screen shots of the backend web-based TFCat annotation tool. Figure S6: Sox-containing cluster membership for the evaluated cluster pruning methods. Figure S7: Fox-containing cluster membership for the evaluated cluster pruning methods. Figure S8: an example of pruned Fox-containing clusters generated using the I's only method using a threshold of 0.21. [file gb-2009-10-3-r29-S1.pdf]
